# WNT5A-ROR2 axis mediates VEGF dependence of BRAF mutant melanoma

**DOI:** 10.1007/s13402-022-00757-7

**Published:** 2022-12-21

**Authors:** Nicholas Coupe, Lina Guo, Esther Bridges, Leticia Campo, Olivia Espinosa, Richard Colling, Andrea Marshall, Ashwin Nandakumar, Ruud van Stiphout, Francesca M. Buffa, Pippa G. Corrie, Mark R. Middleton, Valentine M. Macaulay

**Affiliations:** 1 Department of Oncology, University of Oxford, Old Road Campus Research Building, Oxford, OX3 7DQ UK; 2 Department of Oncology, Oxford Cancer and Haematology Centre, Oxford University Hospitals NHS Foundation Trust, Churchill Hospital, Oxford, OX3 7LJ UK; 3 Department of Cellular Pathology, Oxford University Hospitals NHS Foundation Trust, John Radcliffe Hospital, Oxford, OX3 9DU UK; 4 Warwick Clinical Trials Unit, Division of Health Sciences, University of Warwick, Coventry, UK; 5 Nuffield Department of Surgical Sciences, University of Oxford, Old Road Campus Research Building, Roosevelt Drive, Oxford, OX3 7DQ UK; 6 Department of Oncology, Cambridge University Hospitals NHS Foundation Trust, Addenbrooke’s Hospital, Cambridge, UK

**Keywords:** Melanoma, BRAF, Bevacizumab, ROR2, WNT5A

## Abstract

**Purpose:**

Despite recent advances, approximately 50% of patient with metastatic melanoma eventually succumb to the disease. Patients with melanomas harboring a BRAF mutation (BRAF^Mut^) have a worse prognosis than those with wildtype (BRAF^WT^) tumors. Unexpectedly, interim AVAST-M Phase III trial data reported benefit from adjuvant anti-VEGF bevacizumab only in the BRAF^Mut^ group. We sought to find mechanisms underpinning this sensitivity.

**Methods:**

We investigated this finding *in vitro* and *in vivo* using melanoma cell lines and clones generated by BRAF^V600E^ knock-in on a BRAF^WT^ background.

**Results:**

Compared with BRAF^WT^ cells, isogenic BRAF^V600E^ clones secreted more VEGF and exhibited accelerated growth rates as spheroids and xenografts, which were more vascular and proliferative. Recapitulating AVAST-M findings, bevacizumab affected only BRAF^V600E^ xenografts, inducing significant tumor growth delay, reduced vascularity and increased necrosis. We identified 814 differentially expressed genes in isogenic BRAF^V600E^/BRAF^WT^ clones. Of 61 genes concordantly deregulated in clinical melanomas *ROR2* was one of the most upregulated by BRAF^V600E^. *ROR2* was shown to be RAF-MEK regulated in BRAF^V600E^ cells and its depletion suppressed VEGF secretion down to BRAF^WT^ levels. The ROR2 ligand *WNT5A* was also overexpressed in BRAF^Mut^ melanomas, and in ROR2-overexpressing BRAF^V600E^ cells MEK inhibition downregulated WNT5A and VEGF secretion.

**Conclusions:**

These data implicate WNT5A-ROR2 in VEGF secretion, vascularity, adverse outcomes and bevacizumab sensitivity of BRAF^Mut^ melanomas, suggesting that this axis has potential therapeutic relevance.

**Supplementary Information:**

The online version contains supplementary material available at 10.1007/s13402-022-00757-7.

## Introduction

The incidence of cutaneous melanoma is increasing and, when metastatic, it is lethal in most cases [1]. Melanoma is a vascular tumor, and tumor angiogenesis in primary melanomas correlates with tumor thickness, risk of recurrence and a poor prognosis [2, 3]. The principal angiogenesis mediator is vascular endothelial growth factor (VEGF), which is expressed and secreted as isoforms A-E, with VEGFA being the main driver of pathological angiogenesis [4]. VEGFA has many isoforms including pro-angiogenic splice variants VEGF_121_ and VEGF_165_ that promote endothelial cell proliferation and migration, and anti-angiogenic isoforms [5]. VEGF expression is tightly regulated at multiple levels, one of the best-characterized routes being as a transcriptional target of hypoxia inducible factor HIF-1α, which is stabilized in hypoxia and degraded in normoxia due to von Hippel-Lindau protein (pVHL)-mediated post-translational HIF-1α modification [6, 7]. The tumor contents of VEGFA and VEGF receptor VEGFR2 are higher in malignant melanomas vs benign nevi and in metastatic vs primary melanomas [4, 8]. During melanomagenesis, rapid tumor cell proliferation, especially during the vertical growth phase, increases oxygen and nutrient demands, causing local hypoxia that triggers angiogenesis and vascular remodelling [9]. Increased angiogenesis, measured by intra-tumoral microvessel density (MVD), is known to correlate with disease progression and an adverse prognosis in melanoma patients [10].

Recognition of this role for VEGF represented the driver for the Phase III AVAST-M adjuvant clinical trial [11], which tested the hypothesis that VEGF-driven angiogenesis is required for progression of micro-metastases to metastatic disease. From 2007–2012, the trial recruited 1343 patients with high risk (AJCC 7^th^ defined stage IIB, IIC or III) melanoma. Patients were randomised to 12 months of adjuvant treatment with bevacizumab, or observation, the standard of care at trial initiation. Bevacizumab is a humanized VEGF-neutralizing monoclonal antibody that is reported to have modest activity in patients with advanced melanoma [12]. Interim analysis of the AVAST-M trial reported no difference in overall survival between the two arms, with hazard ratio (HR) 0.97 and 95% confidence intervals (CI) 0.78–1.22 (*p* = 0.76), although patients on the bevacizumab arm experienced significantly greater disease-free survival (DFS) compared to the observation arm (HR 0.83, 95% CI 0.70–0.98, *p* = 0.03). One year after completion of all treatment, pre-planned interim subgroup analysis revealed that DFS prolongation in the bevacizumab arm was solely observed in patients with BRAF^V600E^ mutant melanomas, with HR 0.06 (95% CI 0.43–0.85, *p* = 0.004) compared with HR 0.87 (95% CI 0.64–1.18, *p* = 0.36) in BRAF wild-type (BRAF^WT^) melanoma patients [11].

*BRAF* mutations occur in ~ 50% of cutaneous melanomas, of which > 80% are *BRAF*^*V600E*^; other mutations activating the RAS-RAF-extracellular signal regulated kinase (ERK) pathway include *NRAS* (30% of cases) and *NF1* (14%) mutations, all usually mutually exclusive, activating RAS-RAF-ERK in > 90% of cutaneous melanomas [13]. This pathway is recognized as one of many factors that contribute to transcriptional and post-transcriptional regulation of VEGF expression [6], known to involve HIF-1α through at least 2 mechanisms. Firstly, ERK mediated phosphorylation of 4E-BP1 increases HIF-1α translation and secondly, ERK signalling recruits co-factor p300 to HIF-1α, enhancing the transcription of HIF-1α target genes [14]. HIF-1α expression has been reported to be significantly higher in 30 melanoma cell lines compared with non-transformed melanocyte cell lines, and introduction of BRAF^V600E^ has been found to increases HIF-1α stabilisation, although HIF-1α expression was shown to be suppressed by siRNA-mediated depletion of either WT or mutant BRAF [15].

While previous studies highlighted roles for HIF-1α and VEGF in melanoma angiogenesis [15, 16], there are no previous data on the relative importance of angiogenesis or response to anti-angiogenic therapy in BRAF^WT^ vs. BRAF^Mut^ melanoma models or primary patient tumors. Therefore, the AVAST-M finding of a BRAF-dependent response to anti-angiogenic therapy was unexpected. Here, we report an increased VEGF secretion in BRAF^Mut^ cells, identify transmembrane receptor tyrosine kinase ROR2 and its ligand WNT5A as upregulated in BRAF^V600E^ melanomas, and show that both ROR2 and WNT5A are regulated by RAF-MEK-ERK and play a critical role in the angiogenic profile of BRAF^V600E^ melanomas. These data suggest that targeting this axis merits exploration as therapy for patients with BRAF^V600E^ melanoma.

## Materials and methods

### Cell lines and reagents

Human melanoma cell lines CHL1, SKMEL2 and HCMB were obtained from the American Type Culture Collection. A375P, A375M, 501mel and WM35 were obtained from Professor Colin Goding (Ludwig Institute for Cancer Research, Oxford, UK), ME, Na8 and SKMEL23 from Professor Vincenzo Cerundolo (Weatherall Institute of Molecular Medicine, Oxford, UK) and SKMEL28 and SKMEL29 from Cancer Research UK Cell Services. The cell lines were authenticated at Eurofins Genomics. Human umbilical vein endothelial cells (HUVECs) were obtained from Lonza (#2517A). All cell lines were negative when tested for mycoplasma (MycoAlert kit, Lonza Rockland Inc, Rockland, USA). BRAF^V600E^ knock-in plasmids were from Horizon Discovery, Waterbeach, UK), ROR2 siRNAs from Life Technologies (#AM51331) and Qiagen (# SI00287518) and control siRNA (AllStars negative control) from Qiagen. PLX4720 (Selleck, #S1152), Trametinib (Selleck, #S2673) and Cedirinib (AZD2171, Selleck Chemicals, #S1017) were reconstituted in DMSO at 10 mM. Single use aliquots were stored at -80 °C. ROR2 overexpression plasmid (#RC215640) was from Origene and empty vector pCMV6 (#PS100001) from James Chettle (Department of Oncology, University of Oxford, UK).

### Plasmid and siRNA transfection

For siRNA transfection, cells were seeded to achieve 40% confluence the following day and transfected with Lipofectamine 2000 and OptiMEM (both Life Technologies) using siRNAs at 20 nM final concentration as described in [17]. Cells were harvested 48 h after transfection for further analysis. For stable ROR2 overexpression, cells were seeded in 6 well plates and transfected at 80% confluence. Plasmid DNA (2 mg/well) and Lipofectamine 2000 (6 ml) were mixed with 180 ml OptiMEM, incubated for 20 min at room temperature and added dropwise to wells containing 2 ml fresh DMEM with 10% FCS*.* The following day, medium was replaced with fresh medium plus G418 (700 µg/ml for A375M, 800 µg/ml for CHL1). After 7 days surviving colonies were picked, expanded and screened for ROR2 expression by Western blotting.

### Cell viability, 2D and 3D growth, motility and invasion assays

For viability assays, cells (1000 to 2000 cells/well depending on proliferation rate) were seeded, treated the following day with drugs or solvent control and, after 3 days, viability was measured using a CellTiter-Glo Luminescent Assay (Promega). Cell growth was determined by counting live cells by Trypan Blue exclusion. Spheroids were cultured in Ultra-low adherent round bottom 96-well plates in medium containing 5% Corning Matrigel matrix, and their sizes were quantified using GELCOUNT (Oxford Optronix). For wound-healing and invasion assays cells were used at 80% confluence, scratched (‘wounded’) with a pipette tip and monitored using a xCELLigence® Real-Time Cell Analysis instrument or Live Cell Imaging Analyser IncuCyte ZOOM, calculating relative wound closures using IncuCyte 2011A analysis software.

### Western blotting and VEGF ELISA

Western blotting was performed as reported before [18] using antibodies listed in Appendix Table S1. Conditioned media were collected from cells cultured in a fixed volume of medium with 10% FCS. Total cellular protein was quantified (BCA protein assay) and VEGF ELISA was performed on media and cell lysates using a Quantikine Human VEGF ELISA (R&D). Intracellular VEGF content was expressed as mean ± SEM pg/µg total protein and concentrations in conditioned media were normalized for differences in cell number.

### Reverse transcription-quantitative PCR (RT-qPCR)

RNAs were extracted using a RNeasy Kit (Qiagen) or a PureLink RNA Mini Kit (Life Technologies), reverse transcribed (SuperScript III First-Strand Synthesis SuperMix, Invitrogen) and amplified using a SYBR Select master mix (Life Technologies) and primers listed in Appendix Table S2 on an Applied Biosystems 7500 Real-Time PCR System. Some reactions used a one-step process (Luna Universal One-Step qRT-PCR kit, New England Biolabs). Relative expression levels were calculated using the 2-ΔΔCt method as in [19] and normalized to the housekeeping gene *TUBA6*. Where fold change against a single control was inappropriate Ct values were normalized using the formula: 2-ΔCT (test sample).

### Creation of isogenic BRAF WT/mutant model

BRAF WT CHL1 cells were co-transfected with two plasmids (Horizon Discovery): a *BRAF*-disrupting plasmid containing a chimeric gRNA scaffold and WT Cas9 to introduce a double strand break (DSB) in exon 15 of *BRAF*, and a donor plasmid containing a CMV-driven eGFP selection marker integrated into intron 15, BRAF^V600E^ mutation (1799 T > A) and three silent mutations to prevent donor re-cutting. After 10 days, GFP-positive cells were single-cell sorted (Beckman Coulter Legacy MoFlo MLS High Speed Cell Sorter) and expanded, after which cells were lysed using a DNA release buffer (Microzone #2ML-250) and DNA tested by PCR using two pairs of screening primers and 2 pairs of confirmation primers. Incorporation of correct donor sequences was checked by DNA sequencing and expression of *BRAF* mRNA was assessed using primers listed in Appendix Table S2.

### Microarray-based expression analysis

Triplicate independently collected RNAs from isogenic clones were processed using an Illumina TotalPrep-96 RNA Amplification Kit followed by an Illumina Whole-Genome Gene Expression Direct Hybridisation Assay. Labelled cRNAs were hybridised to Human HT12v4.0 BeadChips. Raw beadchip data were pre-processed using Illumina Inc. GenomeStudio version 1.9.0. and further processed by quantile normalisation and log2 transformation. Genes that were not expressed were filtered out by including only probes with detection *p*-values ≤ 0.05 for at least one sample. The normalised and filtered data were subjected to hierarchical clustering, after which differently expressed genes (DEGs) were identified by LIMMA version 3.19.16 (https://bioconductor.org/packages/release/bioc/html/limma.html), with the necessary contrasts detailed in the results section for each analysis. A false discovery rate (FDR) was corrected using the Benjamini–Hochberg procedure and significantly differentially expressed genes were defined as genes with a FDR < 0.05. Significantly differentially expressed genes were assessed for enriched pathway ontology using the PANTHER database in the GeneCodis pathway http://genecodis.cnb.csic.es/ [20–22].

### *In vivo* experiments, serum and tumor analyses

All *in vivo* work was conducted under UK Home Office approved Project Licence PPL 30 /3197. Before Home Office submission, the Project Licence was approved by the Oxford University Animal Welfare and Ethical Review Board*.* Six-week old female CD1 immunodeficient mice (Charles River) were injected subcutaneously in their flanks with 7.5 × 10^6^ melanoma cells/mouse. When tumors reached ~ 150 mm^3^ (calculated as p/6 × length x width x height) mice were randomised to treatment with 100 µl PBS or 10 mg/kg bevacizumab intraperitoneally three times a week. Mice were sacrificed when tumors reached licence limits (1.44 cm^3^). Blood was obtained by cardiac puncture and sera were assayed for human VEGF using a Human VEGF Quantikine ELISA Kit (R&D Systems). Formalin-fixed paraffin embedded tumors underwent immunohistochemical analysis using a FLEX staining kit (Ailgent) with antibodies directed against Ki67 (M7240; Dako, 1:50), CA9 (M75; BD Biosciences, 1:1000) and CD31 (#550,274; BD Biosciences, 1:50). CA9 and Ki67 were quantified by ImageJ using colour deconvolution as described previously [23]. CD31 was quantified in 20 random fields at × 20 magnification and expressed as average number of vessels per field. Necrosis was quantified on haematoxylin and eosin (H&E) stained slides by Image J (US National Institutes of Health, Bethesda, MD, USA).

### Statistical analysis

All data other than microarray data (see above) were analysed using Microsoft Excel for Mac and GraphPad Prism 8 (GraphPad Software Inc, USA). Statistical significance was determined using student t-test to compare 2 groups, one-way ANOVA for > 2 groups and two-way ANOVA for comparisons between curves. Graphs show mean ± standard error of mean and a *p*-value < 0.05 was considered significant.

## Results

### BRAF^V600E^ knock-in induces a mutant BRAF phenotype and enhances anchorage-independent growth and VEGF secretion

**Fig. 1:**
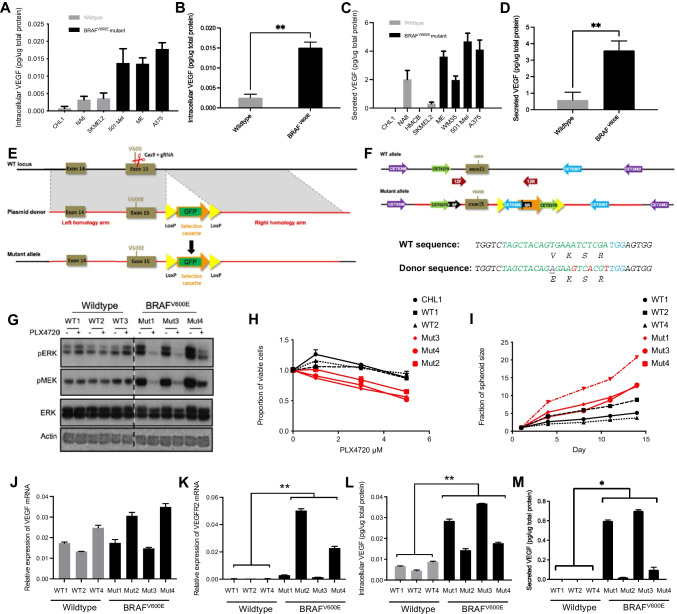
BRAF mutant melanoma cells exhibit accelerated growth in 3D and increased VEGF secretion. **(A-B)** Whole cell extracts and **(C-D)** conditioned media from non-isogenic melanoma cell lines were assayed for VEGF content. Differences between BRAF WT and mutant cell lines was tested by unpaired t-test (**p < 0.01). **(E)** Scheme showing introduction of BRAF^V600E^ mutation into BRAF^WT^ host cells. **(F)** WT sequence replacement with mutant V600E allele, showing upper: sites of screening and confirmation primers (listed in Appendix Table S2); lower: WT and mutant sequences with mutation (purple), silent mutations (red), gRNA guide sequence (green) and PAM site (blue). **(G)** Melanoma cells were treated with 1 µM PLX4720 for 24 h and lysed for Western blotting. BRAF^V600E^ clones exhibited constitutive MAPK pathway activation (dashed line: cropped out lanes) that was inhibited by PLX4720. **(H)** Response to PLX4720 tested in viability assays. Pooled data from 3 independent repeats in parental cells and clones were analyzed by 2-way ANOVA. There was a significant difference (*p* < 0.0001) in the overall response of BRAF WT (black) and BRAF mutant (red) cells to PLX4720. Sidak’s Multiple comparisons test showed that BRAF mutant cells were significantly more sensitive to PLX4720 at 1 µM (*p* < 0.0001), 3 µM (*p* < 0.0001) and 5 µM (*p* < 0.001). **(I)** Growth in anchorage-independent conditions tested in 3D spheroid assays. Results are expressed as mean ± SEM fraction of size at day 1. Pooled data from 3 independent repeats in parental cells and clones were analyzed by 2-way ANOVA, which indicated that BRAF mutant spheroids (red) grew significantly faster (*p* < 0.0001) than BRAF WT spheroids (black). Differences at each time-point were assessed by Sidak’s Multiple comparisons test, showing significant differences at day 4 (*p* < 0.001) and days 8–14 (*p* < 0.0001). **(J)** No significant difference was observed in *VEGF* mRNA expression between BRAF^WT^ and BRAF^V600E^ clones. **(K-M)** The introduced BRAF^V600E^ mutation resulted in *VEGFR2* mRNA upregulation **(K)**, increased intracellular **(L)** and secreted VEGF **(M)** protein (*n* = 3 independent replicates in each case, **p ≤ 0.01, unpaired t-test)

We tested two potential explanations for the differential sensitivity of BRAF^V600E^ melanomas to bevacizumab. First, based on reports that melanoma cells express VEGFR2 receptors [24] we tested for direct inhibitory effects involving autocrine VEGF/VEGFR signalling. Using assays for 2D and 3D growth, we found no evidence that bevacizumab or the VEGFR inhibitor AZD2171 (cediranib) inhibited the growth of human BRAF^V600E^ or BRAF^WT^ melanoma cells (Appendix Fig. S1A-C). We also tested HMCB (BRAF^WT^) and SKMEL28 (BRAF^V600E^) cells using wound-healing assays. While BRAF^V600^ mutant SKMEL28 cells migrated more rapidly than HMCB cells, the phenotype was not affected by bevacizumab (Appendix Fig. S1D). Based on a lack of evidence for direct autocrine effects of bevacizumab on cell proliferation or migration, and given that VEGF is the sole target of bevacizumab, we explored the hypothesis that BRAF mutation-dependent effects of bevacizumab may be mediated indirectly via effects on angiogenesis. First, we tested whether the BRAF status affects VEGF secretion by quantifying intracellular and secreted VEGF through ELISA in a panel of BRAF^V600E^ and BRAF^WT^ melanoma cell lines. The results obtained suggested a greater VEGF content in conditioned media and cell lysates collected from BRAF^V600E^ cells cultured in normoxia (Fig. 1A-D). VEGF was secreted at higher levels in hypoxia as expected, although hypoxic cells showed no differences by BRAF status (Appendix Fig. S1E-F).

Keeping in mind that these results could be confounded by genetic differences between the cell lines, we generated an isogenic model using the triple-negative (*BRAF*/*NRAS*/*NF1* WT) CHL1 cell line [25], introducing BRAF^V600E^ by homologous recombination (Fig. 1E-F). Clones were expanded, screened by PCR (Appendix Fig. S2A), sequenced and tested by RT-qPCR for expression of BRAF mRNA. Compared to the parental CHL1 cell line and BRAF^WT^ CHL1 clones, many of the screened CRISPR clones showed almost complete loss of BRAF^WT^ expression, with variable expression of BRAF^V600E^ mRNA in homozygous BRAF^V600E^ clones (Appendix Fig. S2B-C). Mutant clones showed constitutive activation of the MAPK signalling axis, and in response to vemurafenib analog PLX4720 showed suppression of viability and ERK phosphorylation without changes in ERK expression (Fig. 1G-H, Appendix Fig. S2D), indicating induced sensitivity to BRAF^Mut^-specific inhibition. BRAF^V600E^ mutant clones also showed a more rapid 3D spheroid growth compared with parental CHL1 cultures and WT CHL1 clones, suggesting a more aggressive phenotype under anchorage-independent conditions (Fig. 1I). Next, the effect of introducing *BRAF*^V600E^ on VEGF and VEGFR2 expression and the response to VEGF inhibition was assessed. No change in *VEGF* expression was observed at the mRNA level (Fig. 1J), and a variable *VEGFR2* upregulation at the transcriptional level in 2 out of 4 BRAF^V600E^ clones (Fig. 1K). Of note, we detected significant increases in intracellular VEGF protein content in the mutant clones, with a variable but generally increased VEGF secretion compared with BRAF^WT^ cells (Fig. 1L-M). Despite these changes, melanoma cells were not growth inhibited *in vitro* by the VEGF-neutralizing antibody bevacizumab or the VEGFR inhibitor cediranib (Appendix Fig. S2E-F), reinforcing the notion that bevacizumab is unlikely to exert a direct effect on the melanoma cells themselves.

### Introduction of BRAF^V600E^ enhances growth of highly vascular melanomas and induces sensitivity to bevacizumab *in vivo*

**Fig. 2:**
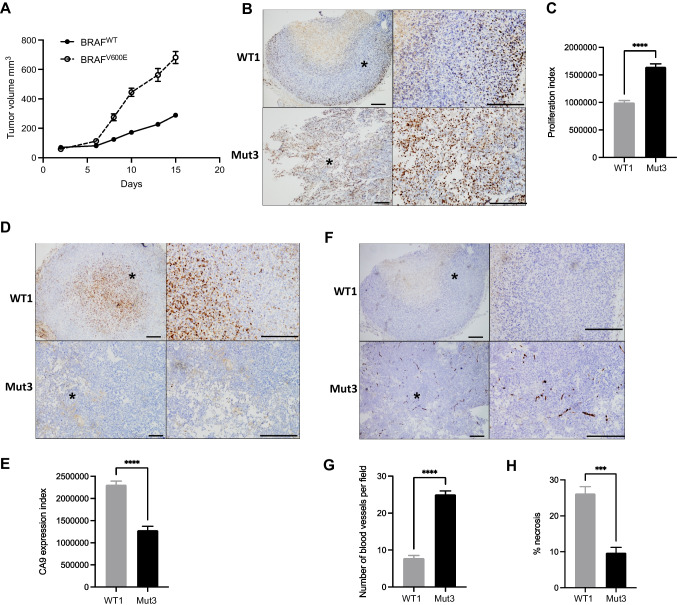
BRAF^V600E^ melanoma xenografts grow more rapidly than isogenic BRAF^WT^ tumors and are more proliferative and vascular. **(A)** Growth of isogenic BRAF^WT^ (WT1) and BRAF^V600E^ (Mut3) xenografts, showing a highly significant difference in tumor growth curves (*p* < 0.0001) by 2-way ANOVA. Sidak’s Multiple comparisons test showed no significant difference up to day 6 and significant differences at each timepoint thereafter (*p* < 0.0001). **(B-G)** Representative images of WT1 and BRAF^V600E^ Mut3 xenografts with quantification of staining for Ki67 **(B-C)**, CA9 **(D-E)** and CD31 positive microvessels **(F-G)**. Asterisks on images to left indicate areas shown at higher magnification on right, scale bars 100 μm. **(H)** Necrosis quantified on H&E sections as necrotic area percentage of total area. Graphs show mean ± SEM, *n* = 5 tumors for all comparisons. ***p < 0.001 ****p < 0.0001

**Fig. 3:**
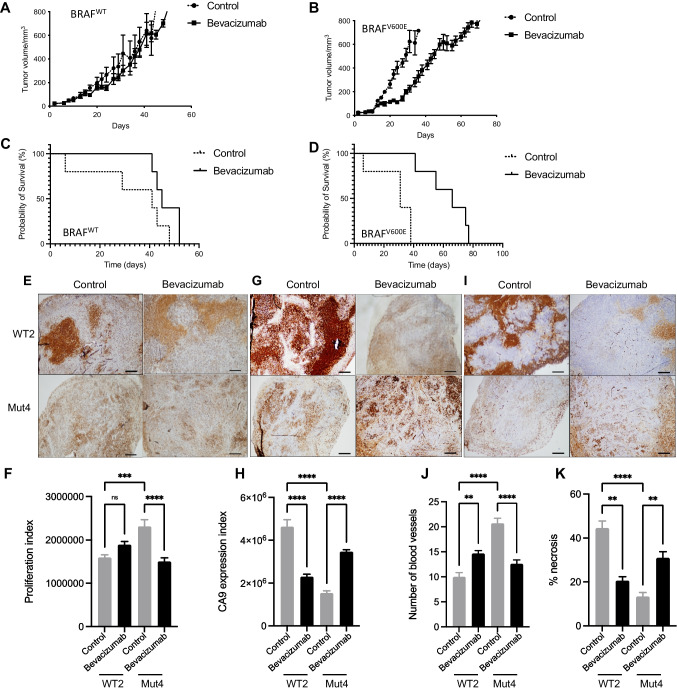
Bevacizumab retards growth of BRAF^V600E^ but not BRAF^WT^ xenografts. Effect of bevacizumab on tumor growth (**A-B**) and survival (**C-D**) in mice engrafted with isogenic BRAF^WT^ or BRAF^V600E^ melanomas. Immunohistochemical evaluation and quantification of Ki67% (**E–F**), CA9 (**G-H**) and CD31 positive blood vessels (**I-J**). C = Control, B = Bevacizumab. Scale bars 100 μm. (**K**) Quantification of necrosis as in Fig. 2H. A-D analysed using 2-way ANOVA. C-D analysed using Log-rank (Mantel-Cox) analysis. F, H, J, H analysed by 1-way ANOVA with multiple comparisons. Graphs show mean ± SEM, *n* = 5 tumors/group, **p ≤ 0.01 ***p ≤ 0.001 ****p ≤ 0.0001

To assess effects of bevacizumab on tumor growth *in vivo*, immunodeficient mice were inoculated with isogenic melanoma cells: two BRAF^WT^clones: WT1 and WT2, and two BRAF^V600E^ clones: Mut3 and Mut4. Mice inoculated with BRAF^V600E^ Mut3 cells developed rapidly growing vascular tumors and had to be sacrificed after 12 days. Mice bearing WT1 xenografts were culled simultaneously for comparative purposes (Fig. 2A). In these treatment-naïve xenografts, BRAF^V600E^ Mut3 tumors had a significantly higher proliferation rate than WT1 tumors as judged by Ki67 index (Fig. 2B-C). Carbonic anhydrase 9 (CA9), a hypoxia marker tightly regulated by HIF/pVHL [26] was significantly higher in BRAF^WT^ xenografts (Fig. 2D-E) suggesting higher levels of hypoxia. Consistent with this observation, CD31 staining confirmed that BRAF^V600E^ melanomas were more vascular with a significant increase in blood vessel density (Fig. 2F-G) and less necrosis than BRAF^WT^ melanomas (Fig. 2H). Mice bearing BRAF^WT^ WT2 and BRAF^V600E^ mutant Mut4 xenografts were randomly allocated to treatment with solvent or bevacizumab. BRAF^V600E^ xenografts showed a growth delay in response to bevacizumab while no such effect was observed in BRAF^WT^ xenografts (Fig. 3A-B). Bevacizumab prolonged the survival of mice harboring *BRAF*^V600E^ (*p* = 0.0026) but not *BRAF*^WT^ tumors (Fig. 3C-D). This result suggests that BRAF^V600E^ knock-in induced the sensitivity to bevacizumab, essentially recapitulating the interim results of the AVAST-M trial [11].

Given that bevacizumab inhibits only human but not murine VEGF [27], a further implication of this result may be that VEGF secretion by human melanoma cells had altered the phenotype, growth rate and bevacizumab response of melanoma xenografts. To investigate this concept, we tested serum for human VEGF, and assessed proliferation, microvasculature and hypoxia/necrosis markers in control and bevacizumab-treated tumors. We were unable to detect human VEGF in any of the treatment groups (data not shown), but we did identify significant differences in tumor tissues. A higher Ki67 expression was observed in control-treated BRAF^V600E^ Mut4 tumors compared to WT2 tumors (Fig. 3E-F). The proliferation index was significantly reduced in the BRAF^V600E^ Mut4 group after bevacizumab treatment (Fig. 3E-F), with no difference in BRAF^WT^ WT2 tumors, paralleling the inhibitory effect on tumor growth (Fig. 3B). CA9 expression was significantly higher in *BRAF*^WT^ WT2 than *BRAF*^V600E^ Mut4 control-treated xenografts (Fig. 3G-H), consistent with the findings in the comparison between WT1 and Mut3 xenografts (Fig. 2C-D). However, following bevacizumab treatment CA9 expression decreased in BRAF^WT^ tumor tissues and increased in BRAF^V600E^ xenografts (Fig. 3G-H). As with the comparison of treatment-naïve tumors (Fig. 2E-F), CD31 staining of microvasculature revealed more vessels in control-treated Mut4 BRAF^V600E^ tumors than in WT2 tumors (Fig. 3I-J). Reflecting changes in CA9 expression, blood vessel numbers were increased by bevacizumab in BRAF^WT^ tumors and reduced in BRAF^V600E^ xenografts (Fig. 3I-J). In control-treated tumors the relative necrotic areas were greater in BRAF^WT^ tumors compared to BRAF^V600E^ mutant tumors and were reduced by bevacizumab in WT2 tumors and increased in mutant Mut4 tumors (Fig. 3K), the latter being consistent with an anti-angiogenic effect. Taken together, these observations suggest that knock-in of the BRAF^V600E^ mutation increased malignant phenotypes that were influenced by tumor-derived VEGF and were suppressed by bevacizumab.

### Transcriptional effects of BRAF^V600E^ knock-in include upregulation of genes with direct and indirect effects on angiogenesis

**Fig. 4:**
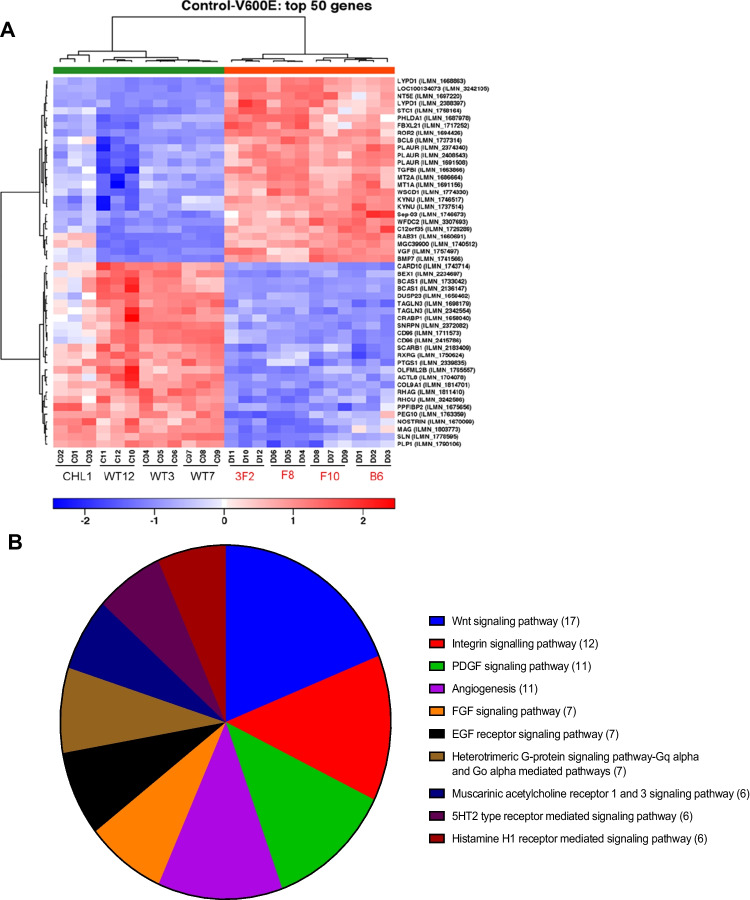
BRAF^V600E^ knock-in influences the expression of genes in growth factor and angiogenesis pathways. (**A**) Expression heatmap of top 50 differentially expressed genes (DEGs) identified in microarray analysis of isogenic BRAF^WT^ and BRAF^V600E^ melanoma clones and parental CHL1 cells. (**B**) Top 10 signaling pathways represented by DEGs in the BRAF^V600E^ and wildtype clones. Numbers in brackets refer to the numbers of genes annotated within each pathway (http://www.pantherdb.org)

Aiming to identify changes in gene expression that may contribute to enhanced VEGF secretion, we performed transcriptional profiling by microarray analysis on RNAs extracted from CHL1 parental cells, three BRAF^WT^ and four BRAF^V600E^ clones. Hierarchical clustering of normalised and filtered data showed clustering of replicate samples and a distinctive distribution of the experimental groups (Appendix Fig. S3A). Compared with BRAF^WT^ clones, introduction of BRAF^V600E^ clearly affected the global gene expression, with 814 significantly differentially expressed genes (DEGs, adjusted *p*-value < 0.05). The top 50 genes showed a distinctive pattern of expression between BRAF^WT^ and BRAF^V600E^ groups (Fig. 4A, Appendix Table S3) and included 11 genes related to angiogenesis pathways (Fig. 4B). *VEGF* itself was not differentially expressed, consistent with an unaltered expression as detected by RT-qPCR (Fig. 1J).

**Fig. 5:**
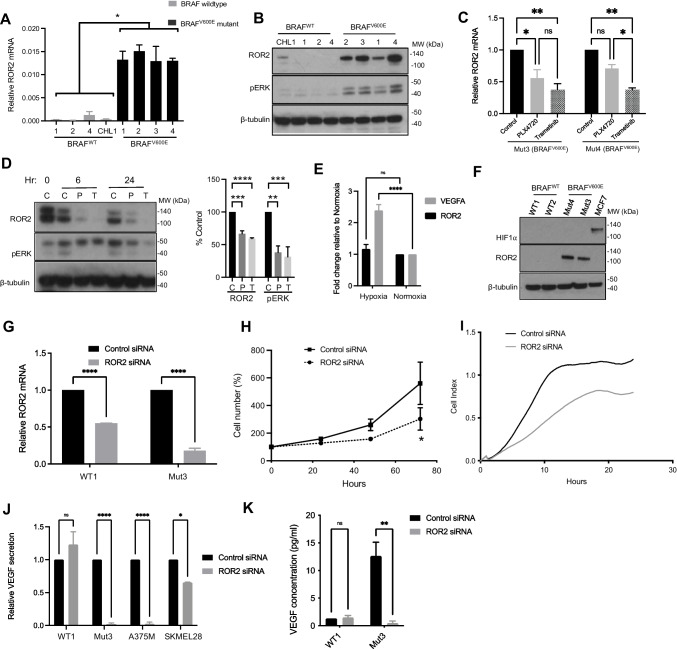
ROR2 upregulation in BRAF^V600E^ melanoma cells promotes VEGF secretion. (**A-B**) CHL1 cells and isogenic clones tested for ROR2 expression at the mRNA (**A**) and protein (**B**) levels by RT-qPCR and Western blotting, respectively. (**C-D**) Clonal BRAF^V600E^ mutant cells were treated for 0, 6 or 24 h hours with solvent (control, C), 2 µm PLX4720 (P) or 5 nM trametinib (T) and *ROR2.* mRNA (**C**) and protein (**D**) were assessed as in A-B. ERK phosphorylation was quantified in 3 independent repeats of D, expressed relative to β-tubulin and shown in graph to right. Differences tested with 1-way ANOVA. (**E**) A375M cells were cultured under normoxia or hypoxia (1% O_2_) for 48 h after which the expression of *VEGFA* and *ROR2* mRNA was quantified by RT-qPCR. (**F**) Western blot for HIF-1⍺ in whole cell extracts of BRAF^V600E^ and BRAF^WT^ melanoma clones cultured in normoxia. MCF7 cells were cultured in 1% O_2_ for 6 h as a positive control for HIF-1⍺ detection. (**G-I**) Clonal BRAF^V600E^ Mut3 cells were transfected with non-silencing or ROR2 siRNA and tested for (**G**) ROR2 expression, (**H**) proliferation and (**I**) migration. (**J-K**) VEGF quantified in medium conditioned by clonal BRAF^WT^ and BRAF^V600E^ cells following control or ROR2 siRNA transfection, expressed as (**J**) ROR2 fold change compared with control transfected WT1 clone, and (**K**) absolute VEGF concentration. For A, BRAF^V600E^ and WT cell lines were pooled and compared by unpaired t-test. C, E, G-K analysed using 2-way ANOVA. Graphs show mean ± SEM from 3 independent assays, *p ≤ 0.05 **p ≤ 0.01 ****p ≤ 0.0001

To ascertain the extent to which this pattern of differential gene expression reflects findings in clinical BRAF^V600^ vs BRAF^WT^ melanomas, we compared genes that were differentially expressed in the isogenic clones with genes differentially expressed in a publicly available melanoma dataset from The Cancer Genome Atlas (TCGA), fire hose legacy. This is a depository of publicly available NCI-generated data (https://www.cbioportal.org/) that includes 287 melanomas of which 134 (47%) harbor a BRAF^V600^ mutation, including BRAF^V600E^ in 121 (41%), BRAF^V600M^ in 17 (6%) and BRAF^V600G^ in 2 (0.7%). From this dataset we identified 2071 DEGs between BRAF mutated (all mutations) and wildtype melanomas (adjusted *p*-values < 0.05; false discoveries corrected using the Benjamini–Hochberg procedure). The top 100 DEGs are listed in Appendix Table S2. By assessing the isogenic clonal model and clinical TCGA datasets together, 96 genes were found to be differentially expressed in both, of which 61 genes (64%) showed concordant differential expression while 35 genes (36%) were not concordant in terms of direction of expression (Appendix Table S3). While a difference is expected given the heterogeneity in sample biology and processing, we focused our validation on genes that were differentially expressed in both datasets and with a potential novel relevance to angiogenesis and VEGF signaling. Differential gene expression was tested in RNAs extracted from BRAF^V600E^ and BRAF^WT^ isogenic clones by RT-qPCR. We confirmed differential expression of *ROR2* (Fig. 5A-B), *IGFBP2* and *FGFR1* (Appendix Fig. S3B-C), all being significantly upregulated in BRAF^V600E^ clones.

### ROR2 is transcriptionally upregulated by BRAF^V600E^ and promotes VEGF secretion in BRAF^V600E^ melanoma cells

Of the three genes validated as upregulated in BRAF^V600E^ vs BRAF^WT^ melanoma, ROR2 was selected for further interrogation because of its striking pattern of upregulation in BRAF^V600E^ clones (Fig. 5A) and contribution to aggressive phenotypes in melanoma [28], which we hypothesized might include regulation of VEGF. First, we confirmed ROR2 overexpression at the protein level in BRAF^V600E^ clones (Fig. 5B). To test associations of this phenotype with activation of cell signaling downstream of the introduced BRAF^V600E^ mutation, we treated isogenic BRAF^V600E^ cells with the pharmacological BRAF^V600E^ inhibitor PLX4720 or MEK inhibitor trametinib. Both agents suppressed ROR2 expression at both the mRNA and protein level (Fig. 5C-D, Appendix Fig. S3D), supporting the hypothesis that ROR2 upregulation results from signalling activation induced by the BRAF^V600E^ mutation. Following its stabilization under hypoxia, HIF-1 α is a well -characterized driver of VEGF upregulation [29]. The transcriptional activity of HIF-1α may also be enhanced by other factors including oncogenic BRAF [30]. Given that ROR2 expression reportedly undergoes HIF-1α -mediated upregulation in hypoxia [31], we tested whether increased ROR2 expression and VEGF secretion in BRAF^V600E^ melanoma cells resulted from HIF-1α stabilization. In A375M cells that harbor endogenous BRAF^V600E^, 48 h culture under hypoxia (1% oxygen) led to upregulation of *VEGF* mRNA as expected, but ROR2 was unaffected (Fig. 5E). In the isogenic BRAF^V600E^ and BRAF^WT^ melanoma clones cultured under normoxia, differential ROR2 expression was again apparent, but HIF-1α protein was not detectable, suggesting that BRAF^V600E^ acquisition did not promote ROR2 upregulation via HIF-1α stabilization (Fig. 5F). These data are consistent with MEK-ERK-dependent, hypoxia-independent ROR2 upregulation in BRAF^V600E^ melanoma cells.

Next, we used gene silencing to explore phenotypes associated with ROR2 and the impact of the BRAF^V600E^ mutation on these phenotypes. ROR2 depletion inhibited the proliferation of BRAF^V600E^ but not BRAF^WT^ cells (Fig. 5H, Appendix Fig. S3E-F). ROR2-depleted BRAF^V600E^ cells were also less migratory and less invasive, compared with control-transfected cells (Fig. 5I, Appendix Fig. S3G). Differences in migration and invasion were observed within 20 h, suggesting that these effects were not influenced by proliferation changes, which were apparent only after 24 h (Fig. 5H). Although it would have been preferable to confirm the effects on proliferation, migration and invasion using a second independent siRNA, our results are consistent with the pro-migratory, pro-invasive phenotype reported for ROR2 in melanoma, ovarian and renal cancer cells [28, 32, 33], and suggest that ROR2 is functioning as predicted in the context of the introduced BRAF^V600E^ mutation.

We next interrogated the contribution of ROR2 to VEGF expression at the level of transcription, intracellular protein content and secretion. ROR2 depletion inhibited *VEGF* mRNA expression in isogenic BRAF^V600E^ melanoma cells, but not in isogenic BRAF^WT^ cells, nor in A375M and SKMEL28 cells that harbour an endogenous BRAF^V600E^ mutation (Appendix Fig. S4A). Indeed, *VEGF* mRNA was apparently upregulated by ROR2 depletion in SKMEL28 cells. Quantification of intracellular VEGF showed a reduction upon ROR2 depletion in isogenic BRAF^V600E^ melanoma cells, but no significant change in isogenic BRAF^WT^ cells or A375M and SKMEL28 cells (Appendix Fig. S4B). Next, VEGF secretion was tested in culture medium conditioned by melanoma cells transfected with ROR2 or control siRNA. No change was observed in the low levels of secreted VEGF in isogenic BRAF^WT^ cells upon ROR2 depletion, while in contrast there was consistent suppression of VEGF secretion in ROR2-depleted BRAF mutant cells, including BRAF^V600E^ isogenic cells and A375M and SKMEL28 cells (Fig. 5J). The inability of ROR2 siRNA to suppress *VEGF* mRNA expression in BRAF WT clones and A375 cells could be attributable in part to the relatively low *ROR2* expression in these cells, but is more likely to reflect a predominant effect at the level of VEGF secretion. Indeed, effects on the latter were confirmed in A375M cells transfected with a second ROR2 siRNA (Appendix Fig. S4C). When results in the isogenic cells were compared in terms of absolute amounts of VEGF in conditioned medium, it was apparent that ROR2 knockdown resulted in a reduced VEGF secretion in BRAF^V600E^ isogenic cells to levels comparable to those in the isogenic BRAF^WT^ cells (Fig. 5J, right), suggesting that ROR2 was the main driver of the increase in secreted VEGF.

### Secretion of bioactive VEGF is enhanced by ROR2 in WNT5A-high BRAF^V600E^ melanoma cells

**Fig. 6:**
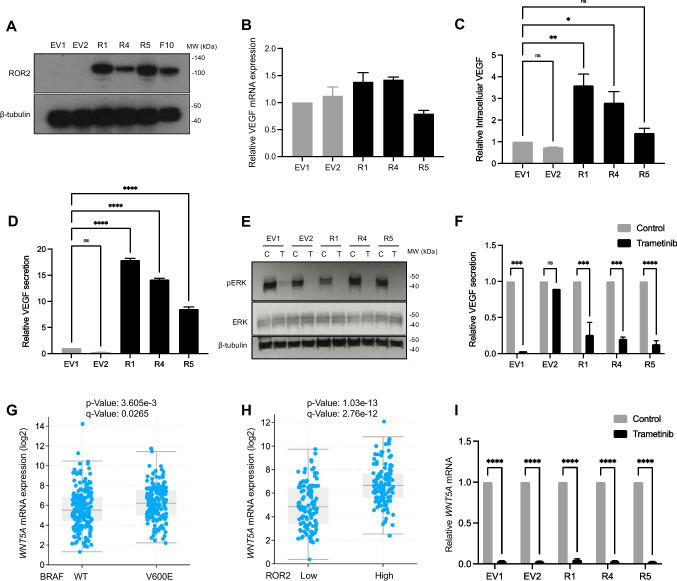
WNT5A and ROR2 are overexpressed in BRAF^V600E^ melanomas, regulated by RAF-MEK-ERK signaling and promote VEGF secretion. (**A**) Western blot for ROR2 in A375M cells stably transfected with empty vector (EV) or ROR2 cDNA (R). (**B-D**). Effect of ROR2 overexpression on VEGF mRNA expression (**B**), intracellular content (**C**) and secretion (**D**). (**E–F**) ROR2 overexpressing and empty vector clones were treated with 5 nM trametinib (T) or solvent control (C) for 24 h and assayed by Western blotting to confirm MEK-ERK inhibition (**E**) and ELISA for VEGF content of conditioned medium (**F**). (**G-H**) Association of *WNT5A* expression with BRAF status (**G**) and *ROR2* expression (**H**) in TCGA firehose legacy melanoma dataset. (**I**) ROR2 and EV clones were treated as in E–F and analysed by RT-qPCR for *WNT5A* mRNA. Data in B-D, F and I expressed as fold change relative to EV1. *p < 0.05 **p < 0.01 ***p < 0.001 ****p < 0.0001

ROR2 phenotypes were further explored by overexpressing ROR2 in CHL1 cells, the BRAF WT parental cell line from which the isogenic clones were generated. We found that ROR2 overexpression in CHL1 cells did not induce any consistent changes in motility or invasion through matrigel (Appendix Fig. S4D-F) nor any increase in VEGF secretion, which remained undetectable (not shown). The putative ligand for ROR2 is WNT5A [28] and it was, therefore, suspected that the lack of phenotype was related to the absence of ligand. We interrogated data from the Cancer Cell Line Encyclopedia (CCLE, https://depmap.org/portal/download/) and identified several BRAF WT melanoma cell lines with a relatively high *WNT5A* expression (Appendix Fig. S4G). However, none was available to us and there were no data on *ROR2* or *WNT5A* in CHL1 cells. CCLE data indicated relatively low *ROR2* and high *WNT5A* expression in A375 cells that harbor endogenous BRAF^V600E^. Using RT-qPCR we confirmed that *WNT5A* was clearly detectable in A375M cells but undetectable in CHL1 cells (Appendix Fig. S4H). Furthermore, A375M cells appeared to exhibit relatively low *ROR2* levels (Appendix Fig. S4G), suggesting that this cell line may serve as an appropriate host in which to explore effects of ROR2 overexpression. Therefore, we stably transfected *ROR2* cDNA into the A375M cell line. Transfected clones were confirmed to upregulate ROR2 at the mRNA and protein levels, comparable to ROR2 expression in the isogenic BRAF^V600E^ Mut3 clone (Fig. 6A). *VEGF* transcription was unaffected by ROR2 overexpression (Fig. 6B), consistent with the absence of an effect on *VEGF* mRNA upon ROR2-depletion of A375M cells shown earlier (Appendix Fig. S4A). ROR2 overexpression modestly, but significantly, upregulated intracellular VEGF protein and induced a more significant increase in VEGF secretion (Fig. 6C-D). These data support a role for ROR2 in post-transcriptional VEGF regulation.

Finally, we explored the possibility that VEGF regulation may be driven by an interaction between ROR2 and a putative BRAF^V600E^ driven ligand. ROR2 over-expressing A375M clones were treated with the MEK inhibitor trametinib after which conditioned media were collected at 24 h, at which point minimal cell death was apparent as indicated by a lack of non-viable floating cells. The adherent monolayer was harvested for cell counting and protein isolation to confirm suppression of the MAPK signalling pathway by Western blotting (Fig. 6E). Trametinib did not suppress ROR2 expression (Appendix Fig. S4I), as expected given that ROR2 was under control of a constitutive promoter. However, trametinib did suppress VEGF secretion in ROR2-overexpressing clones and one control clone (Fig. 6F). This ROR2-independent effect on VEGF secretion suggests the involvement of other factor(s) under the influence of the RAF-MEK-ERK pathway. We speculated that this could be WNT5A, given its recognition as a ROR2 ligand [28]. WNT5A was not identified as a DEG in the isogenic clones, and was not differentially expressed in BRAF mutant vs WT cell lines in the CCLE (Appendix Fig. S4G) or among the top 100 DEGs in the TCGA firehose legacy dataset (Appendix Table S2). However, re-interrogation of the latter revealed overexpression of *WNT5A* in BRAF mutant melanomas and a positive association between *WNT5A* and *ROR2* mRNA expression (Fig. 6G-H). Supporting regulation by MEK-ERK, *WNT5A* mRNA expression was suppressed by > 95% following trametinib treatment of both control and ROR2 overexpressing clones (Fig. 6I). These findings suggest that both ROR2 and WNT5A are BRAF-MEK regulated, and that both may be necessary to promote VEGF secretion in BRAF^V600E^ mutant melanomas.

## Discussion

The interim data presented in the AVAST-M trial suggested that BRAF mutant melanomas may be selectively sensitive to bevacizumab. This observation raised the hypothesis that BRAF mutant melanomas may be more dependent on VEGF for their survival and, therefore, vulnerable to treatments inhibiting this factor. Associations between the BRAF^V600E^ mutation and angiogenesis have been reported, including data showing that mutant BRAF^V600E^ knockdown may downregulate HIF-1α protein and decrease viability under hypoxic conditions [15]. Transfection of endothelial cells with mutant BRAF has been reported to increase VEGF secretion, while in melanoma cells pharmacological BRAF inhibition promoted vascular stabilisation via a presumed decrease in aberrant angiogenesis [34]. However, neither of these studies directly compared BRAF mutant vs WT phenotypes or included clinical samples. Although an autocrine VEGF/VEGFR axis has been implicated in melanoma growth [35], we found no evidence that bevacizumab directly inhibits BRAF^V600E^ mutant melanomas. In a panel of non-isogenic lines, we found preliminary evidence of increased VEGF secretion in the BRAF mutant cell lines. This initial result prompted us to generate an isogenic model to explore links between BRAF^V600E^, VEGF secretion and bevacizumab response. The introduced BRAF^V600E^ mutation appeared to mediate more rapid 3D growth, consistent with the more aggressive clinical behaviour of BRAF^V600E^ mutant melanoma relative to WT as observed in the AVAST-M clinical trial [36, 37].

We used this isogenic model to test the hypothesis whether the bevacizumab sensitivity of BRAF^V600E^ melanomas is mediated indirectly via effects on VEGF. Indeed, we found evidence that the introduced BRAF^V600E^ mutation induced an increase in VEGF expression at the protein level, with variable changes in intracellular VEGF content and consistent increase in VEGF secretion *in vitro*. These changes were independent of changes in VEGF or HIF-1α levels, despite published associations between BRAF^V600E^ and HIF-1α expression and the important role of HIF-1α as a well characterised driver of VEGF expression [38]. We next attempted to obtain direct *in vivo* evidence of differential VEGF secretion by assaying serum from tumor-bearing mice. However none was detected, suggesting that the amount of xenograft-derived human VEGF reaching the circulation was below the lower limit of detection of the human VEGF ELISA assay. None-the-less, it is plausible that intra-tumoral concentrations were sufficient to induce a biological response. In support of this notion, a study in nasopharyngeal carcinoma found that human VEGF levels were 30—60 times higher in xenografts than in serum of tumor-bearing mice [39]. Consistent with effects of increased VEGF secretion [40, 41], BRAF^V600E^ xenografts were less hypoxic, proliferated more and contained more CD31 positive blood vessels than isogenic BRAF^WT^ tumors. Importantly, our data also showed that BRAF^V600E^ tumors were more sensitive to bevacizumab, responding with significant tumor growth delay, increased hypoxia and reduction in vascularity. These findings are consistent with the hypothesis that BRAF^V600E^ melanomas are more dependent on VEGF, essentially explaining and recapitulating the interim findings from the AVAST-M trial [11]. We noted that bevacizumab treatment of BRAF^WT^ tumors resulted in a paradoxical increase in vessel counts and reduction in necrosis. We speculate that this apparent pro-vascular response to bevacizumab could reflect very low levels of human VEGF in BRAF^WT^ tumors, promoting greater dependency on other compensatory pro-angiogenic mechanisms, such as the recruitment of stromal pro-angiogenic cells including pro-angiogenic bone-marrow-derived cells, macrophages or activated cancer-associated fibroblasts [42–46].

We acknowledge that the isogenic model we generated was based on introduction of oncogenic BRAF into the already transformed BRAF^WT^ CHL1 cell line. In a pathological context, BRAF mutant melanomas demonstrate ‘oncogene addiction’ to the mutation [47] and a relative paucity of other oncogenic mutations [13]. Therefore, it is plausible that the phenotype was impacted by pre-existing endogenous oncogenic mutations that influenced the final phenotype. However, supporting clinical relevance, we identified a significant overlap of differentially expressed genes in the isogenic BRAF^V600E^/BRAF^WT^ clones and melanoma data from the TCGA (firehose) dataset. Analysing genes differentially expressed in both datasets revealed differential expression of genes associated with angiogenesis, although VEGF itself was not differentially expressed at the mRNA level, consistent with our findings in isogenic clones.

The orphan receptor ROR2 was highly differentially expressed in both the BRAF^V600E^/^WT^ isogenic cell line model and TCGA (firehose legacy) datasets. ROR2 is a component of the non-canonical WNT pathway [48], which has been implicated in the phenomenon of dynamic phenotype switching in which melanomas switch from a highly proliferative, non-invasive phenotype to a phenotype associated with invasion, increased motility and high metastatic potential [28, 31]. A shift from canonical to non-canonical WNT signalling has been reported to drive this phenotype switch and although ROR2 has been implicated in this process [31], to our knowledge, it has not been implicated in processes directly relevant to angiogenesis. Our data generated by ROR2 depletion or overexpression suggest that ROR2 exerts a post-transcriptional control over VEGF secretion. This finding could be tested further using genome editing to delete ROR2. This would enable assessment of the effects of long-term ROR2 loss, to further evaluate the extent to which ROR2 is required for the vascular phenotype and VEGF dependency of BRAF mutant melanoma. We also found evidence that ROR2 phenotypes require concurrent expression of WNT5A. This ligand has not been described previously as a transcriptional target of BRAF^V600E^ induced MAPK signalling and was not identified here as being overexpressed in BRAF^V600E^ melanomas, although we were able to demonstrate an association in a publicly available dataset from the TCGA. However, WNT5A may itself be a transcriptional target of BRAF^V600E^, given our finding that its expression was decreased after MEK inhibition. These data suggest that as a secreted ligand [49], WNT5A may bind and activate ROR2 to result in the BRAF^V600E^-dependent secretory VEGF phenotype we identified. Of note, multiple reports have associated WNT5A with BRAF^V600E^ resistance [31, 50, 51]. BRAF mutant melanoma cell lines resistant to the BRAF^V600E^ inhibitor PLX4720 have been shown to upregulate WNT5A, and their sensitivity was restored by WNT5A depletion [51]. While multiple mechanisms of BRAF inhibitor resistance have been described, a fundamental mechanism remains re-activation of the MAPK signalling axis [52]. The upregulation of WNT5A in BRAF inhibitor resistant melanomas could feasibly result from MAPK pathway reactivation, which would support an association between WNT5A and BRAF^V600E^ signalling activity.

The mature AVAST-M trial data presented in 2018 reported persisting disease-free survival benefit in the bevacizumab arm (HR 0.85; 95% CI 0.74–0.99, *p* = 0.03), although this did not translate into an improvement in overall survival (HR 0.98; CI 0.82–1.16, *p* = 0.78). Patients with BRAF mutant melanomas treated with bevacizumab no longer exhibited significantly improved disease-free survival (HR 0.81 95% CI 0.60–1.10) nor overall survival (HR 0.80; 95% CI 0.57–1.13, *p* = 0.21) rates [36]. Lack of persisting benefit suggests that that over the longer term, compensatory mechanisms may mitigate the benefit demonstrated in the earlier interim analysis [11]. In a broader context, melanoma treatment has progressed considerably in recent years. The median survival for metastatic disease approaches 3 years and the risk of recurrence following resection of high-risk melanoma has approximately halved [53–56]. However, recurrence after resection of stage IIIC melanoma still approaches 40% and metastatic melanoma remains incurable, with a need for new therapeutic approaches. A novel antibody–drug conjugate targeting ROR2 (CAB-ROR2-ADC) is currently in a Phase I/II study [57]Given the known roles of ROR2 in phenotype switching and invasion in melanoma [28, 58, 59] and the data we present here revealing a role in VEGF secretion in BRAF^V600E^ melanomas, we propose that the WNT-ROR2 axis represents an attractive treatment target in patients with BRAF mutant melanoma.

## Supplementary Information

Below is the link to the electronic supplementary material.

Supplementary file1 (PPTX 2215 KB)

## Data Availability

Authors can confirm that all relevant data are included in the article and/or its supplementary information files. Any additional data (particularly that from the microarray analysis) can be made available on upon request.

## Abbreviations

*BRAF*^*Mut*^: BRAF mutant
*BRAF*^*WT*^: BRAF wildtype
*VEGF*: Vascular endothelial growth factor
*pVHL*: Von Hippel-Lindau protein
*VEGFR2*: Vascular endothelial growth factor receptor
*MVD*: Micro vascular density
*AJCC*: American joint committee on cancer
*DFS*: Disease free survival
*ERK*: Extracellular signal regulated kinase
*HIF-1α*: Hypoxia inducible factor 1 alpha
*CA9*: Carbonic anhydrase 9
*RT-PCR*: Reverse transcription polymerase chain reaction
*TCGA*: The cancer genome atlas
*DEGs*: Differentially expressed genes
