# Supplementary material for: WNT5A-ROR2 axis mediates VEGF dependence of BRAF mutant melanoma
Source: Cell Oncol (Dordr). 2022 Dec 21;46(2):391–407. doi: 10.1007/s13402-022-00757-7 (PMC10060292; doi:10.1007/s13402-022-00757-7)
Supplement: Supplementary file 1 — Supplementary file1 (PPTX 2215 KB) [file 13402_2022_757_MOESM1_ESM.pptx]

## Slide 1
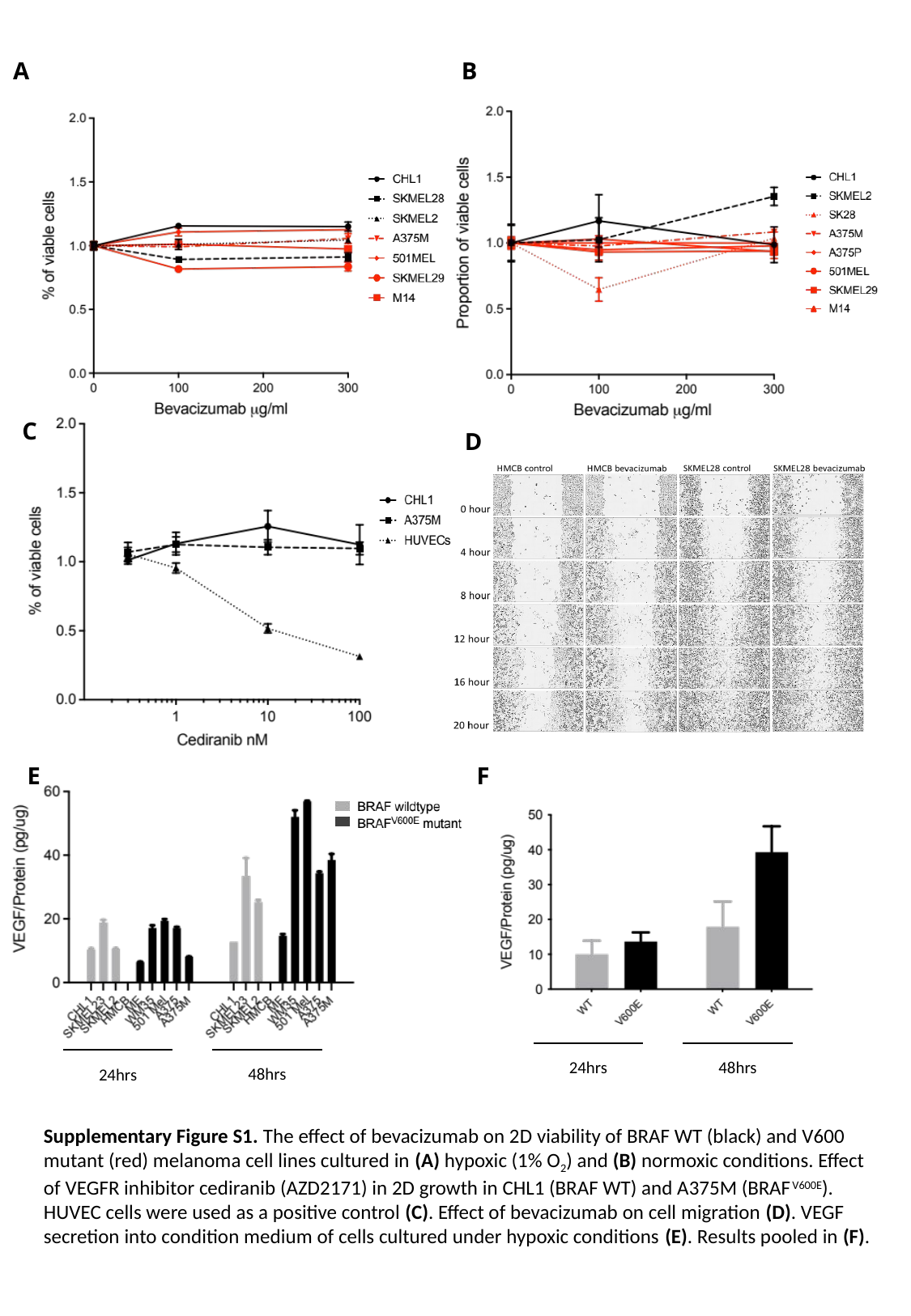

A
B
C
D
E
F
48hrs
24hrs
48hrs
24hrs
Supplementary Figure S1. The effect of bevacizumab on 2D viability of BRAF WT (black) and V600 mutant (red) melanoma cell lines cultured in (A) hypoxic (1% O2) and (B) normoxic conditions. Effect of VEGFR inhibitor cediranib (AZD2171) in 2D growth in CHL1 (BRAF WT) and A375M (BRAFV600E). HUVEC cells were used as a positive control (C). Effect of bevacizumab on cell migration (D). VEGF secretion into condition medium of cells cultured under hypoxic conditions (E). Results pooled in (F).

## Slide 2
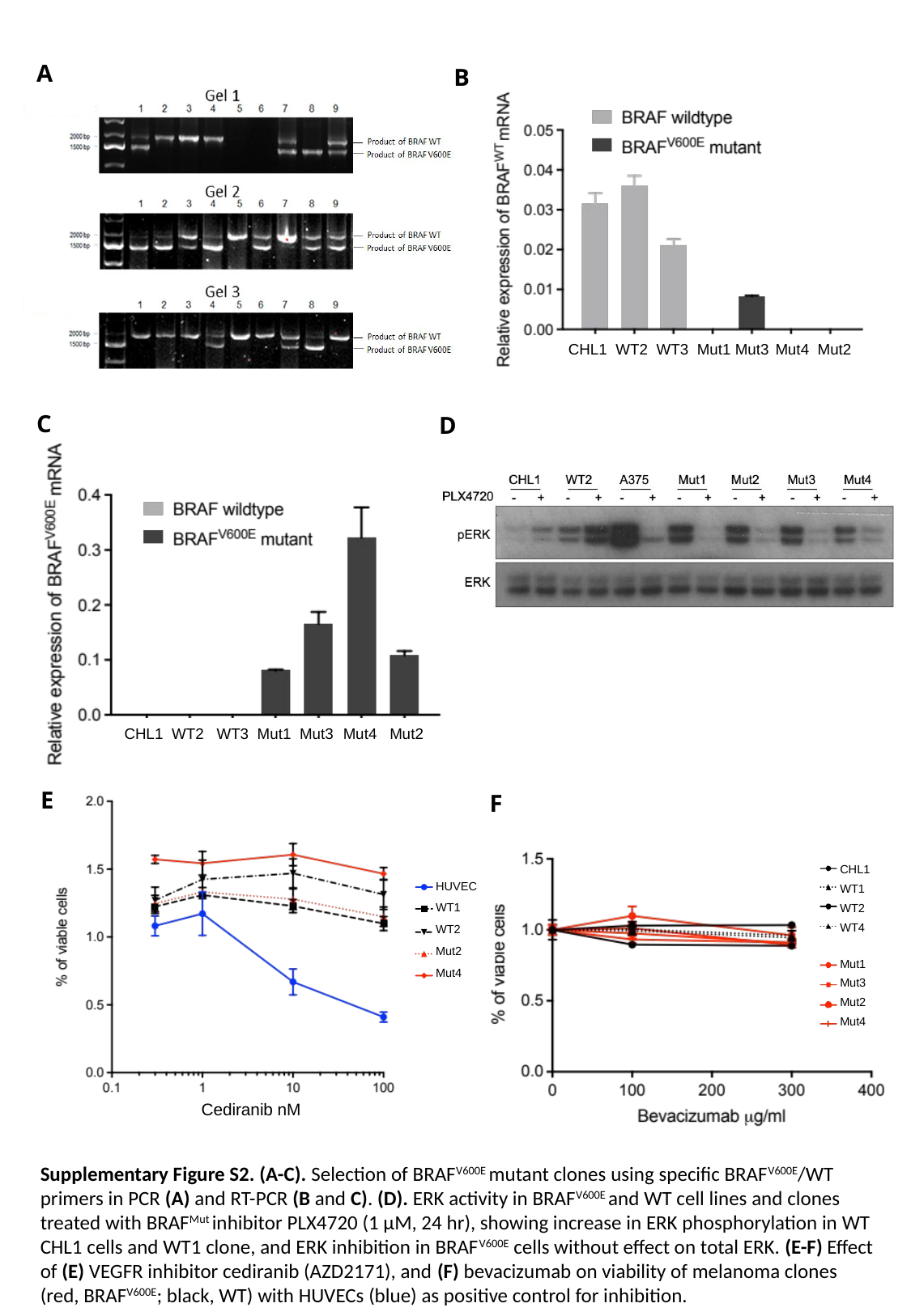

A
B
CHL1 WT2 WT3 Mut1 Mut3 Mut4 Mut2
C
D
CHL1 WT2 WT3 Mut1 Mut3 Mut4 Mut2
E
F
HUVEC
WT1
WT2
Mut2
Mut4
Cediranib nM
CHL1
WT1
WT2
WT4
Mut1
Mut3
Mut2
Mut4
Supplementary Figure S2. (A-C). Selection of BRAFV600E mutant clones using specific BRAFV600E/WT primers in PCR (A) and RT-PCR (B and C). (D). ERK activity in BRAFV600E and WT cell lines and clones treated with BRAFMut inhibitor PLX4720 (1 µM, 24 hr), showing increase in ERK phosphorylation in WT CHL1 cells and WT1 clone, and ERK inhibition in BRAFV600E cells without effect on total ERK. (E-F) Effect of (E) VEGFR inhibitor cediranib (AZD2171), and (F) bevacizumab ​on viability of melanoma clones (red, BRAFV600E; black, WT) with HUVECs (blue) as positive control for inhibition.

## Slide 3
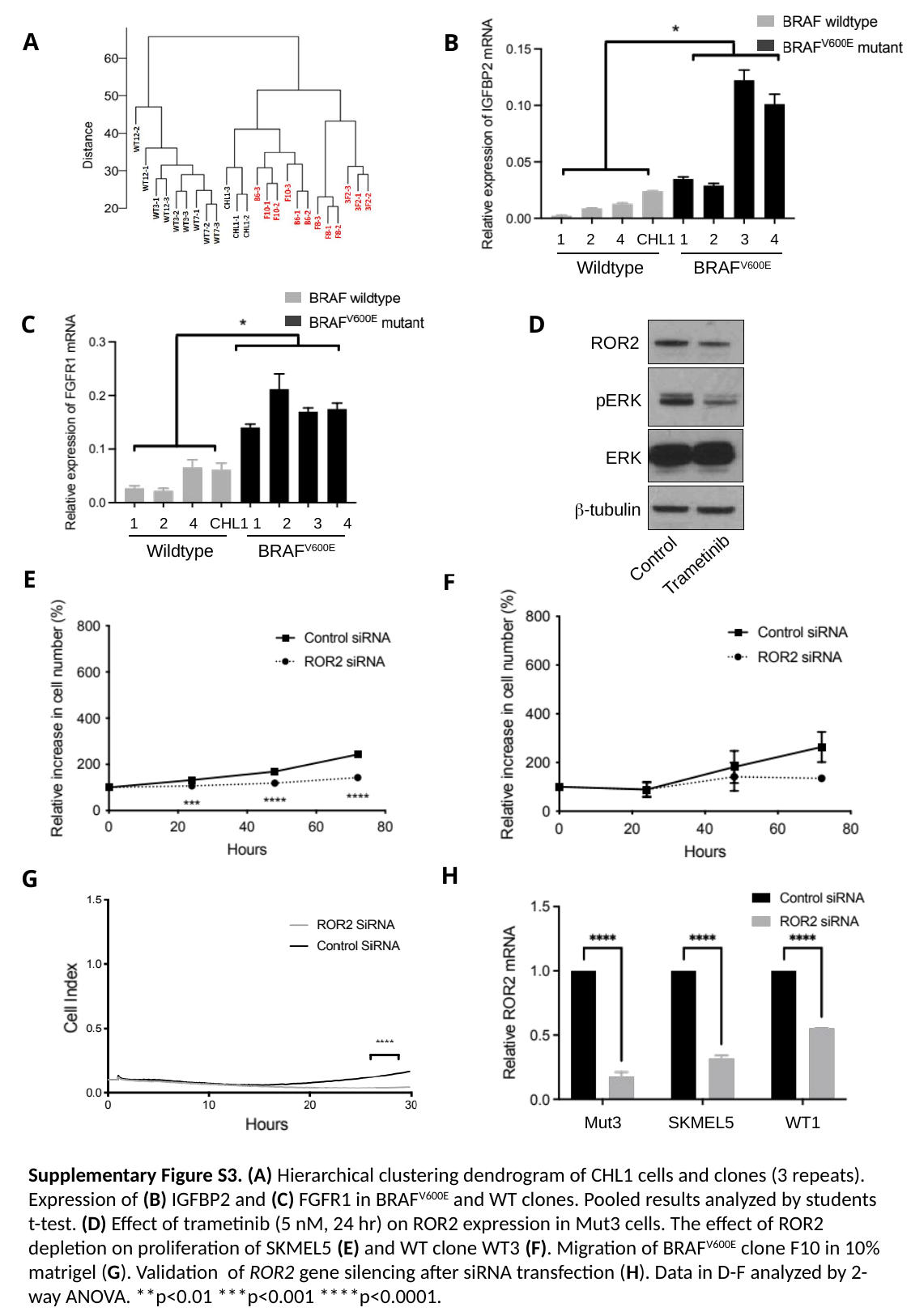

B
 1 2 4 CHL1 1 2 3 4
Wildtype
BRAFV600E
A
 1 2 4 CHL1 1 2 3 4
Wildtype
BRAFV600E
D
C
ROR2
pERK
ERK
b-tubulin
Control
Trametinib
E
F
G
 Mut3 SKMEL5 WT1
H
Supplementary Figure S3. (A) Hierarchical clustering dendrogram of CHL1 cells and clones (3 repeats). Expression of (B) IGFBP2 and (C) FGFR1 in BRAFV600E and WT clones. Pooled results analyzed by students t-test. (D) Effect of trametinib (5 nM, 24 hr) on ROR2 expression in Mut3 cells. The effect of ROR2 depletion on proliferation of SKMEL5 (E) and WT clone WT3 (F). Migration of BRAFV600E clone F10 in 10% matrigel (G). Validation of ROR2 gene silencing after siRNA transfection (H). Data in D-F analyzed by 2-way ANOVA. **p<0.01 ***p<0.001 ****p<0.0001.

## Slide 4
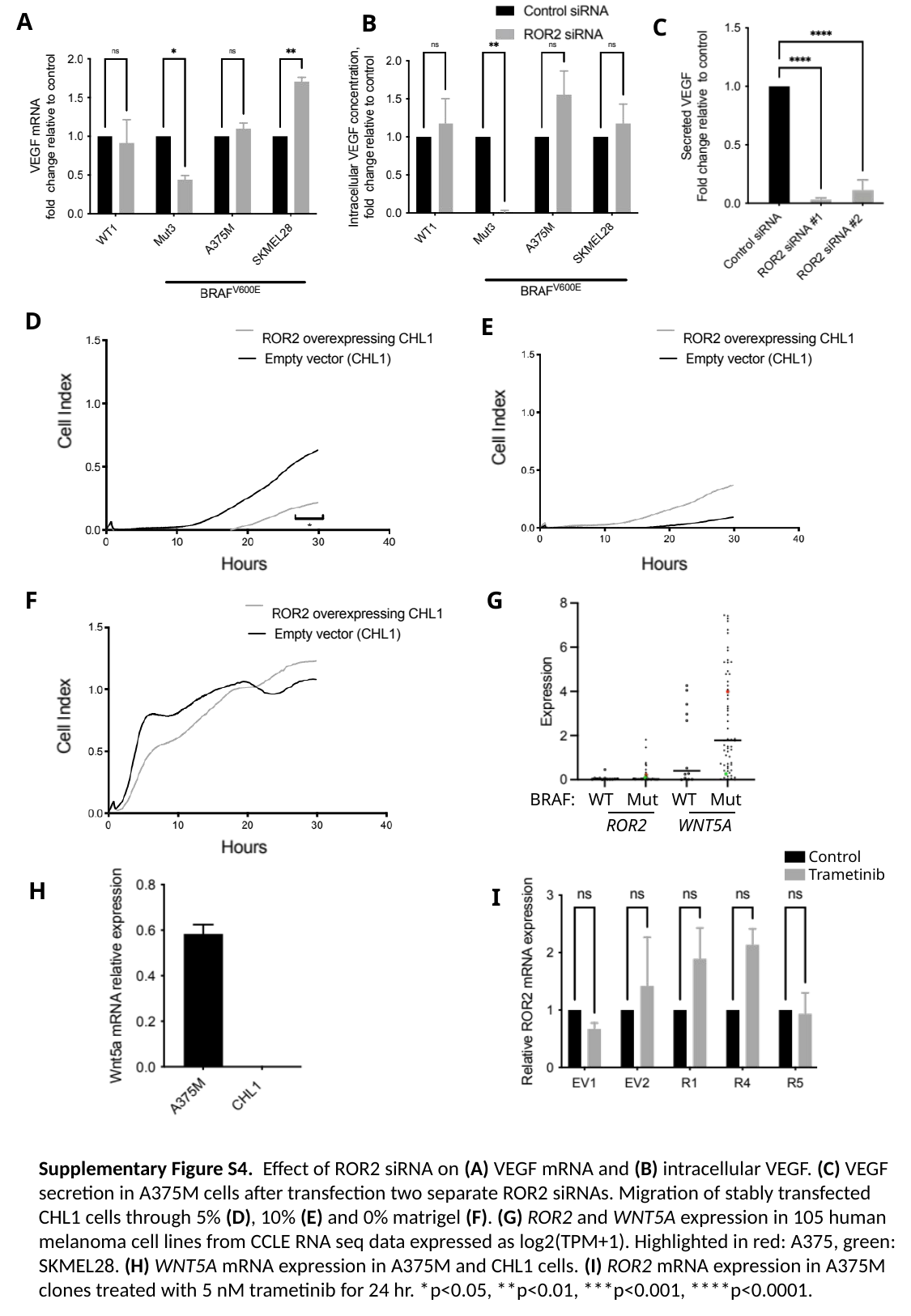

C
A
B
D
E
F
G
BRAF: WT Mut WT Mut
ROR2 WNT5A
Control
Trametinib
H
I
Supplementary Figure S4. Effect of ROR2 siRNA on (A) VEGF mRNA and (B) intracellular VEGF. (C) VEGF secretion in A375M cells after transfection two separate ROR2 siRNAs. Migration of stably transfected CHL1 cells through 5% (D), 10% (E) and 0% matrigel (F). (G) ROR2 and WNT5A expression in 105 human melanoma cell lines from CCLE RNA seq data expressed as log2(TPM+1). Highlighted in red: A375, green: SKMEL28. (H) WNT5A mRNA expression in A375M and CHL1 cells. (I) ROR2 mRNA expression in A375M clones treated with 5 nM trametinib for 24 hr. *p<0.05, **p<0.01, ***p<0.001, ****p<0.0001.

## Slide 5
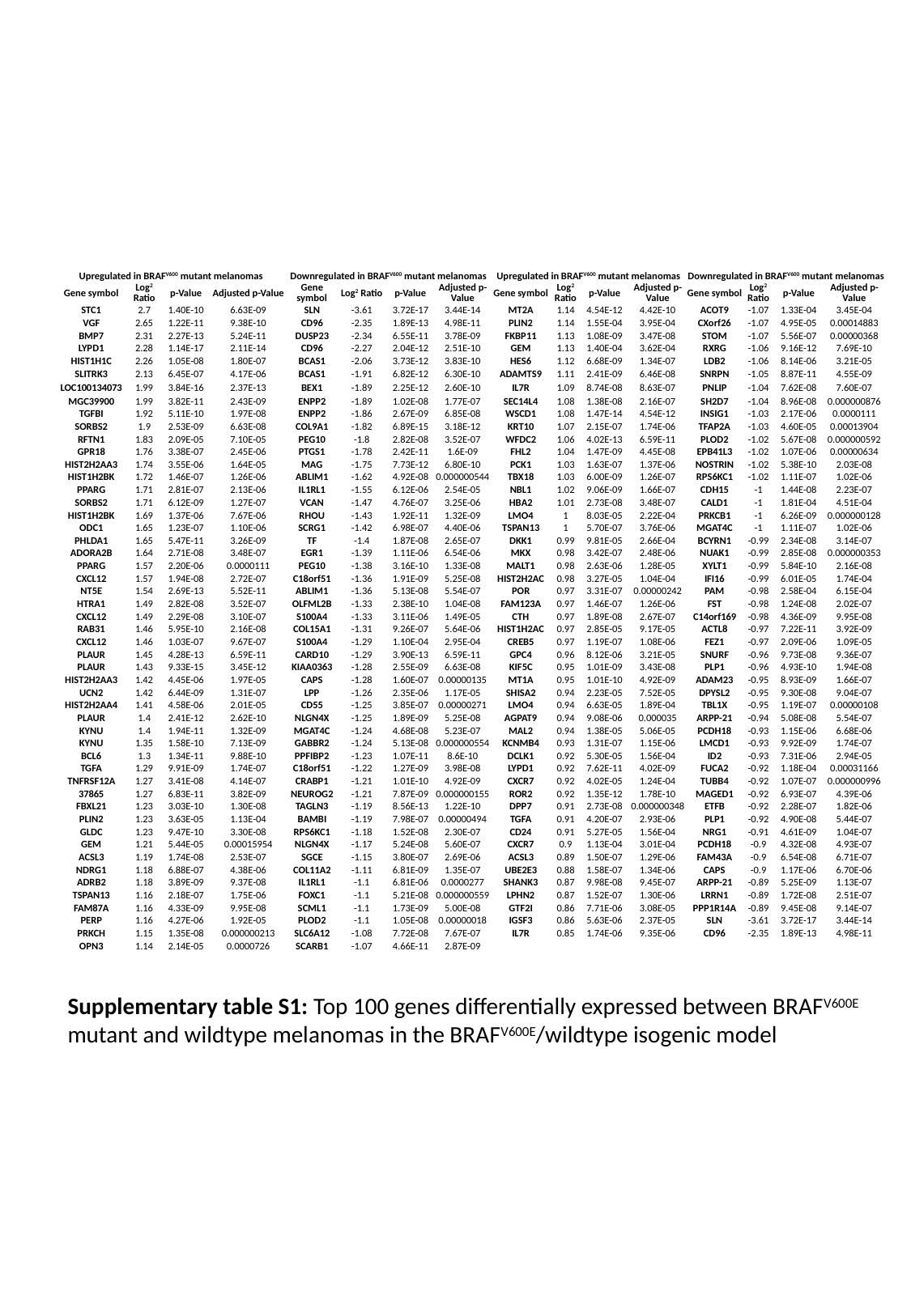

| Upregulated in BRAFV600 mutant melanomas | | | | Downregulated in BRAFV600 mutant melanomas | | | | Upregulated in BRAFV600 mutant melanomas | | | | Downregulated in BRAFV600 mutant melanomas | | | |
| --- | --- | --- | --- | --- | --- | --- | --- | --- | --- | --- | --- | --- | --- | --- | --- |
| Gene symbol | Log2 Ratio | p-Value | Adjusted p-Value | Gene symbol | Log2 Ratio | p-Value | Adjusted p-Value | Gene symbol | Log2 Ratio | p-Value | Adjusted p-Value | Gene symbol | Log2 Ratio | p-Value | Adjusted p-Value |
| STC1 | 2.7 | 1.40E-10 | 6.63E-09 | SLN | -3.61 | 3.72E-17 | 3.44E-14 | MT2A | 1.14 | 4.54E-12 | 4.42E-10 | ACOT9 | -1.07 | 1.33E-04 | 3.45E-04 |
| VGF | 2.65 | 1.22E-11 | 9.38E-10 | CD96 | -2.35 | 1.89E-13 | 4.98E-11 | PLIN2 | 1.14 | 1.55E-04 | 3.95E-04 | CXorf26 | -1.07 | 4.95E-05 | 0.00014883 |
| BMP7 | 2.31 | 2.27E-13 | 5.24E-11 | DUSP23 | -2.34 | 6.55E-11 | 3.78E-09 | FKBP11 | 1.13 | 1.08E-09 | 3.47E-08 | STOM | -1.07 | 5.56E-07 | 0.00000368 |
| LYPD1 | 2.28 | 1.14E-17 | 2.11E-14 | CD96 | -2.27 | 2.04E-12 | 2.51E-10 | GEM | 1.13 | 1.40E-04 | 3.62E-04 | RXRG | -1.06 | 9.16E-12 | 7.69E-10 |
| HIST1H1C | 2.26 | 1.05E-08 | 1.80E-07 | BCAS1 | -2.06 | 3.73E-12 | 3.83E-10 | HES6 | 1.12 | 6.68E-09 | 1.34E-07 | LDB2 | -1.06 | 8.14E-06 | 3.21E-05 |
| SLITRK3 | 2.13 | 6.45E-07 | 4.17E-06 | BCAS1 | -1.91 | 6.82E-12 | 6.30E-10 | ADAMTS9 | 1.11 | 2.41E-09 | 6.46E-08 | SNRPN | -1.05 | 8.87E-11 | 4.55E-09 |
| LOC100134073 | 1.99 | 3.84E-16 | 2.37E-13 | BEX1 | -1.89 | 2.25E-12 | 2.60E-10 | IL7R | 1.09 | 8.74E-08 | 8.63E-07 | PNLIP | -1.04 | 7.62E-08 | 7.60E-07 |
| MGC39900 | 1.99 | 3.82E-11 | 2.43E-09 | ENPP2 | -1.89 | 1.02E-08 | 1.77E-07 | SEC14L4 | 1.08 | 1.38E-08 | 2.16E-07 | SH2D7 | -1.04 | 8.96E-08 | 0.000000876 |
| TGFBI | 1.92 | 5.11E-10 | 1.97E-08 | ENPP2 | -1.86 | 2.67E-09 | 6.85E-08 | WSCD1 | 1.08 | 1.47E-14 | 4.54E-12 | INSIG1 | -1.03 | 2.17E-06 | 0.0000111 |
| SORBS2 | 1.9 | 2.53E-09 | 6.63E-08 | COL9A1 | -1.82 | 6.89E-15 | 3.18E-12 | KRT10 | 1.07 | 2.15E-07 | 1.74E-06 | TFAP2A | -1.03 | 4.60E-05 | 0.00013904 |
| RFTN1 | 1.83 | 2.09E-05 | 7.10E-05 | PEG10 | -1.8 | 2.82E-08 | 3.52E-07 | WFDC2 | 1.06 | 4.02E-13 | 6.59E-11 | PLOD2 | -1.02 | 5.67E-08 | 0.000000592 |
| GPR18 | 1.76 | 3.38E-07 | 2.45E-06 | PTGS1 | -1.78 | 2.42E-11 | 1.6E-09 | FHL2 | 1.04 | 1.47E-09 | 4.45E-08 | EPB41L3 | -1.02 | 1.07E-06 | 0.00000634 |
| HIST2H2AA3 | 1.74 | 3.55E-06 | 1.64E-05 | MAG | -1.75 | 7.73E-12 | 6.80E-10 | PCK1 | 1.03 | 1.63E-07 | 1.37E-06 | NOSTRIN | -1.02 | 5.38E-10 | 2.03E-08 |
| HIST1H2BK | 1.72 | 1.46E-07 | 1.26E-06 | ABLIM1 | -1.62 | 4.92E-08 | 0.000000544 | TBX18 | 1.03 | 6.00E-09 | 1.26E-07 | RPS6KC1 | -1.02 | 1.11E-07 | 1.02E-06 |
| PPARG | 1.71 | 2.81E-07 | 2.13E-06 | IL1RL1 | -1.55 | 6.12E-06 | 2.54E-05 | NBL1 | 1.02 | 9.06E-09 | 1.66E-07 | CDH15 | -1 | 1.44E-08 | 2.23E-07 |
| SORBS2 | 1.71 | 6.12E-09 | 1.27E-07 | VCAN | -1.47 | 4.76E-07 | 3.25E-06 | HBA2 | 1.01 | 2.73E-08 | 3.48E-07 | CALD1 | -1 | 1.81E-04 | 4.51E-04 |
| HIST1H2BK | 1.69 | 1.37E-06 | 7.67E-06 | RHOU | -1.43 | 1.92E-11 | 1.32E-09 | LMO4 | 1 | 8.03E-05 | 2.22E-04 | PRKCB1 | -1 | 6.26E-09 | 0.000000128 |
| ODC1 | 1.65 | 1.23E-07 | 1.10E-06 | SCRG1 | -1.42 | 6.98E-07 | 4.40E-06 | TSPAN13 | 1 | 5.70E-07 | 3.76E-06 | MGAT4C | -1 | 1.11E-07 | 1.02E-06 |
| PHLDA1 | 1.65 | 5.47E-11 | 3.26E-09 | TF | -1.4 | 1.87E-08 | 2.65E-07 | DKK1 | 0.99 | 9.81E-05 | 2.66E-04 | BCYRN1 | -0.99 | 2.34E-08 | 3.14E-07 |
| ADORA2B | 1.64 | 2.71E-08 | 3.48E-07 | EGR1 | -1.39 | 1.11E-06 | 6.54E-06 | MKX | 0.98 | 3.42E-07 | 2.48E-06 | NUAK1 | -0.99 | 2.85E-08 | 0.000000353 |
| PPARG | 1.57 | 2.20E-06 | 0.0000111 | PEG10 | -1.38 | 3.16E-10 | 1.33E-08 | MALT1 | 0.98 | 2.63E-06 | 1.28E-05 | XYLT1 | -0.99 | 5.84E-10 | 2.16E-08 |
| CXCL12 | 1.57 | 1.94E-08 | 2.72E-07 | C18orf51 | -1.36 | 1.91E-09 | 5.25E-08 | HIST2H2AC | 0.98 | 3.27E-05 | 1.04E-04 | IFI16 | -0.99 | 6.01E-05 | 1.74E-04 |
| NT5E | 1.54 | 2.69E-13 | 5.52E-11 | ABLIM1 | -1.36 | 5.13E-08 | 5.54E-07 | POR | 0.97 | 3.31E-07 | 0.00000242 | PAM | -0.98 | 2.58E-04 | 6.15E-04 |
| HTRA1 | 1.49 | 2.82E-08 | 3.52E-07 | OLFML2B | -1.33 | 2.38E-10 | 1.04E-08 | FAM123A | 0.97 | 1.46E-07 | 1.26E-06 | FST | -0.98 | 1.24E-08 | 2.02E-07 |
| CXCL12 | 1.49 | 2.29E-08 | 3.10E-07 | S100A4 | -1.33 | 3.11E-06 | 1.49E-05 | CTH | 0.97 | 1.89E-08 | 2.67E-07 | C14orf169 | -0.98 | 4.36E-09 | 9.95E-08 |
| RAB31 | 1.46 | 5.95E-10 | 2.16E-08 | COL15A1 | -1.31 | 9.26E-07 | 5.64E-06 | HIST1H2AC | 0.97 | 2.85E-05 | 9.17E-05 | ACTL8 | -0.97 | 7.22E-11 | 3.92E-09 |
| CXCL12 | 1.46 | 1.03E-07 | 9.67E-07 | S100A4 | -1.29 | 1.10E-04 | 2.95E-04 | CREB5 | 0.97 | 1.19E-07 | 1.08E-06 | FEZ1 | -0.97 | 2.09E-06 | 1.09E-05 |
| PLAUR | 1.45 | 4.28E-13 | 6.59E-11 | CARD10 | -1.29 | 3.90E-13 | 6.59E-11 | GPC4 | 0.96 | 8.12E-06 | 3.21E-05 | SNURF | -0.96 | 9.73E-08 | 9.36E-07 |
| PLAUR | 1.43 | 9.33E-15 | 3.45E-12 | KIAA0363 | -1.28 | 2.55E-09 | 6.63E-08 | KIF5C | 0.95 | 1.01E-09 | 3.43E-08 | PLP1 | -0.96 | 4.93E-10 | 1.94E-08 |
| HIST2H2AA3 | 1.42 | 4.45E-06 | 1.97E-05 | CAPS | -1.28 | 1.60E-07 | 0.00000135 | MT1A | 0.95 | 1.01E-10 | 4.92E-09 | ADAM23 | -0.95 | 8.93E-09 | 1.66E-07 |
| UCN2 | 1.42 | 6.44E-09 | 1.31E-07 | LPP | -1.26 | 2.35E-06 | 1.17E-05 | SHISA2 | 0.94 | 2.23E-05 | 7.52E-05 | DPYSL2 | -0.95 | 9.30E-08 | 9.04E-07 |
| HIST2H2AA4 | 1.41 | 4.58E-06 | 2.01E-05 | CD55 | -1.25 | 3.85E-07 | 0.00000271 | LMO4 | 0.94 | 6.63E-05 | 1.89E-04 | TBL1X | -0.95 | 1.19E-07 | 0.00000108 |
| PLAUR | 1.4 | 2.41E-12 | 2.62E-10 | NLGN4X | -1.25 | 1.89E-09 | 5.25E-08 | AGPAT9 | 0.94 | 9.08E-06 | 0.000035 | ARPP-21 | -0.94 | 5.08E-08 | 5.54E-07 |
| KYNU | 1.4 | 1.94E-11 | 1.32E-09 | MGAT4C | -1.24 | 4.68E-08 | 5.23E-07 | MAL2 | 0.94 | 1.38E-05 | 5.06E-05 | PCDH18 | -0.93 | 1.15E-06 | 6.68E-06 |
| KYNU | 1.35 | 1.58E-10 | 7.13E-09 | GABBR2 | -1.24 | 5.13E-08 | 0.000000554 | KCNMB4 | 0.93 | 1.31E-07 | 1.15E-06 | LMCD1 | -0.93 | 9.92E-09 | 1.74E-07 |
| BCL6 | 1.3 | 1.34E-11 | 9.88E-10 | PPFIBP2 | -1.23 | 1.07E-11 | 8.6E-10 | DCLK1 | 0.92 | 5.30E-05 | 1.56E-04 | ID2 | -0.93 | 7.31E-06 | 2.94E-05 |
| TGFA | 1.29 | 9.91E-09 | 1.74E-07 | C18orf51 | -1.22 | 1.27E-09 | 3.98E-08 | LYPD1 | 0.92 | 7.62E-11 | 4.02E-09 | FUCA2 | -0.92 | 1.18E-04 | 0.00031166 |
| TNFRSF12A | 1.27 | 3.41E-08 | 4.14E-07 | CRABP1 | -1.21 | 1.01E-10 | 4.92E-09 | CXCR7 | 0.92 | 4.02E-05 | 1.24E-04 | TUBB4 | -0.92 | 1.07E-07 | 0.000000996 |
| 37865 | 1.27 | 6.83E-11 | 3.82E-09 | NEUROG2 | -1.21 | 7.87E-09 | 0.000000155 | ROR2 | 0.92 | 1.35E-12 | 1.78E-10 | MAGED1 | -0.92 | 6.93E-07 | 4.39E-06 |
| FBXL21 | 1.23 | 3.03E-10 | 1.30E-08 | TAGLN3 | -1.19 | 8.56E-13 | 1.22E-10 | DPP7 | 0.91 | 2.73E-08 | 0.000000348 | ETFB | -0.92 | 2.28E-07 | 1.82E-06 |
| PLIN2 | 1.23 | 3.63E-05 | 1.13E-04 | BAMBI | -1.19 | 7.98E-07 | 0.00000494 | TGFA | 0.91 | 4.20E-07 | 2.93E-06 | PLP1 | -0.92 | 4.90E-08 | 5.44E-07 |
| GLDC | 1.23 | 9.47E-10 | 3.30E-08 | RPS6KC1 | -1.18 | 1.52E-08 | 2.30E-07 | CD24 | 0.91 | 5.27E-05 | 1.56E-04 | NRG1 | -0.91 | 4.61E-09 | 1.04E-07 |
| GEM | 1.21 | 5.44E-05 | 0.00015954 | NLGN4X | -1.17 | 5.24E-08 | 5.60E-07 | CXCR7 | 0.9 | 1.13E-04 | 3.01E-04 | PCDH18 | -0.9 | 4.32E-08 | 4.93E-07 |
| ACSL3 | 1.19 | 1.74E-08 | 2.53E-07 | SGCE | -1.15 | 3.80E-07 | 2.69E-06 | ACSL3 | 0.89 | 1.50E-07 | 1.29E-06 | FAM43A | -0.9 | 6.54E-08 | 6.71E-07 |
| NDRG1 | 1.18 | 6.88E-07 | 4.38E-06 | COL11A2 | -1.11 | 6.81E-09 | 1.35E-07 | UBE2E3 | 0.88 | 1.58E-07 | 1.34E-06 | CAPS | -0.9 | 1.17E-06 | 6.70E-06 |
| ADRB2 | 1.18 | 3.89E-09 | 9.37E-08 | IL1RL1 | -1.1 | 6.81E-06 | 0.0000277 | SHANK3 | 0.87 | 9.98E-08 | 9.45E-07 | ARPP-21 | -0.89 | 5.25E-09 | 1.13E-07 |
| TSPAN13 | 1.16 | 2.18E-07 | 1.75E-06 | FOXC1 | -1.1 | 5.21E-08 | 0.000000559 | LPHN2 | 0.87 | 1.52E-07 | 1.30E-06 | LRRN1 | -0.89 | 1.72E-08 | 2.51E-07 |
| FAM87A | 1.16 | 4.33E-09 | 9.95E-08 | SCML1 | -1.1 | 1.73E-09 | 5.00E-08 | GTF2I | 0.86 | 7.71E-06 | 3.08E-05 | PPP1R14A | -0.89 | 9.45E-08 | 9.14E-07 |
| PERP | 1.16 | 4.27E-06 | 1.92E-05 | PLOD2 | -1.1 | 1.05E-08 | 0.00000018 | IGSF3 | 0.86 | 5.63E-06 | 2.37E-05 | SLN | -3.61 | 3.72E-17 | 3.44E-14 |
| PRKCH | 1.15 | 1.35E-08 | 0.000000213 | SLC6A12 | -1.08 | 7.72E-08 | 7.67E-07 | IL7R | 0.85 | 1.74E-06 | 9.35E-06 | CD96 | -2.35 | 1.89E-13 | 4.98E-11 |
| OPN3 | 1.14 | 2.14E-05 | 0.0000726 | SCARB1 | -1.07 | 4.66E-11 | 2.87E-09 | | | | | | | | |
Supplementary table S1: Top 100 genes differentially expressed between BRAFV600E mutant and wildtype melanomas in the BRAFV600E/wildtype isogenic model

## Slide 6
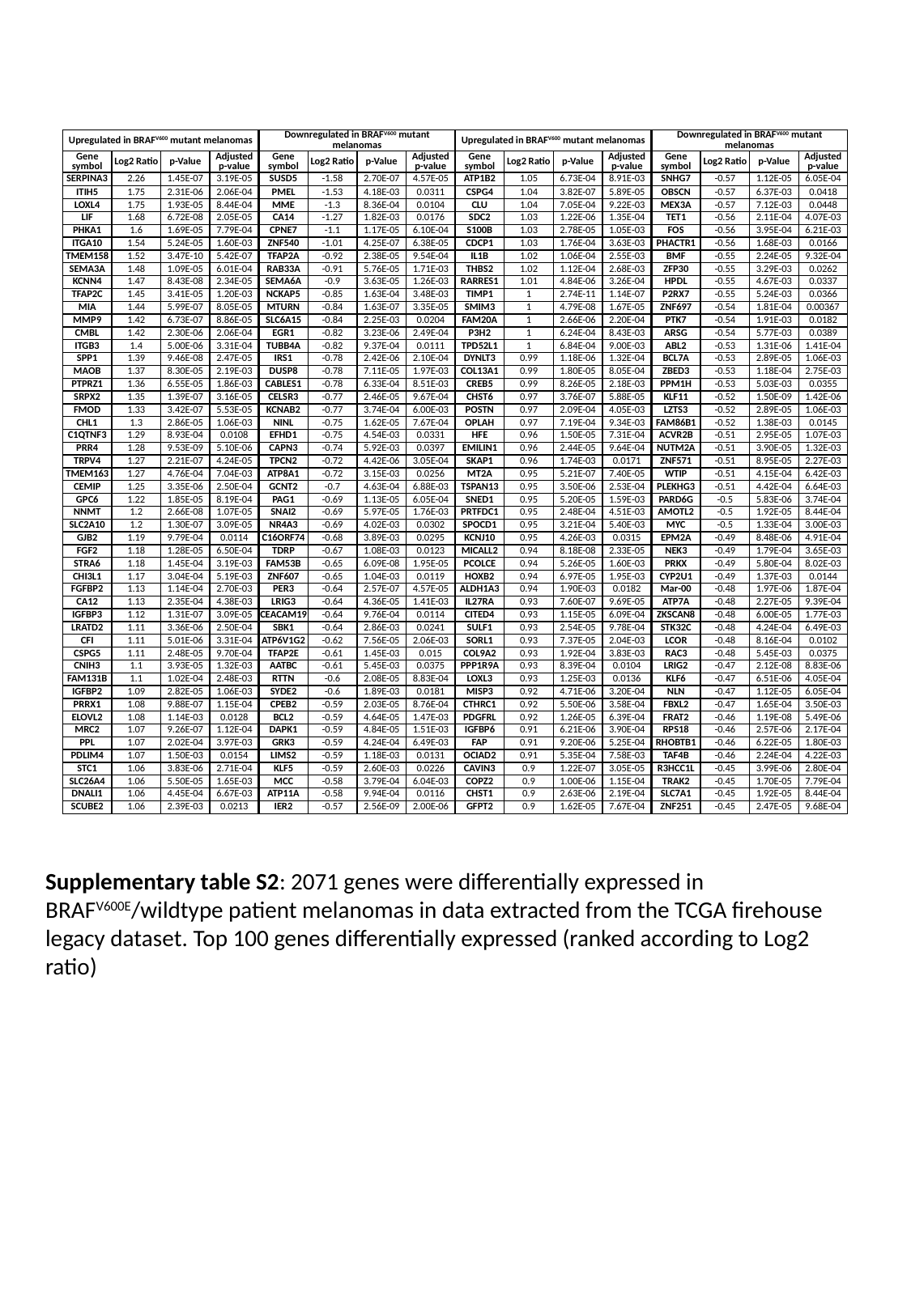

| Upregulated in BRAFV600 mutant melanomas | | | | Downregulated in BRAFV600 mutant melanomas | | | | Upregulated in BRAFV600 mutant melanomas | | | | Downregulated in BRAFV600 mutant melanomas | | | |
| --- | --- | --- | --- | --- | --- | --- | --- | --- | --- | --- | --- | --- | --- | --- | --- |
| Gene symbol | Log2 Ratio | p-Value | Adjusted p-value | Gene symbol | Log2 Ratio | p-Value | Adjusted p-value | Gene symbol | Log2 Ratio | p-Value | Adjusted p-value | Gene symbol | Log2 Ratio | p-Value | Adjusted p-value |
| SERPINA3 | 2.26 | 1.45E-07 | 3.19E-05 | SUSD5 | -1.58 | 2.70E-07 | 4.57E-05 | ATP1B2 | 1.05 | 6.73E-04 | 8.91E-03 | SNHG7 | -0.57 | 1.12E-05 | 6.05E-04 |
| ITIH5 | 1.75 | 2.31E-06 | 2.06E-04 | PMEL | -1.53 | 4.18E-03 | 0.0311 | CSPG4 | 1.04 | 3.82E-07 | 5.89E-05 | OBSCN | -0.57 | 6.37E-03 | 0.0418 |
| LOXL4 | 1.75 | 1.93E-05 | 8.44E-04 | MME | -1.3 | 8.36E-04 | 0.0104 | CLU | 1.04 | 7.05E-04 | 9.22E-03 | MEX3A | -0.57 | 7.12E-03 | 0.0448 |
| LIF | 1.68 | 6.72E-08 | 2.05E-05 | CA14 | -1.27 | 1.82E-03 | 0.0176 | SDC2 | 1.03 | 1.22E-06 | 1.35E-04 | TET1 | -0.56 | 2.11E-04 | 4.07E-03 |
| PHKA1 | 1.6 | 1.69E-05 | 7.79E-04 | CPNE7 | -1.1 | 1.17E-05 | 6.10E-04 | S100B | 1.03 | 2.78E-05 | 1.05E-03 | FOS | -0.56 | 3.95E-04 | 6.21E-03 |
| ITGA10 | 1.54 | 5.24E-05 | 1.60E-03 | ZNF540 | -1.01 | 4.25E-07 | 6.38E-05 | CDCP1 | 1.03 | 1.76E-04 | 3.63E-03 | PHACTR1 | -0.56 | 1.68E-03 | 0.0166 |
| TMEM158 | 1.52 | 3.47E-10 | 5.42E-07 | TFAP2A | -0.92 | 2.38E-05 | 9.54E-04 | IL1B | 1.02 | 1.06E-04 | 2.55E-03 | BMF | -0.55 | 2.24E-05 | 9.32E-04 |
| SEMA3A | 1.48 | 1.09E-05 | 6.01E-04 | RAB33A | -0.91 | 5.76E-05 | 1.71E-03 | THBS2 | 1.02 | 1.12E-04 | 2.68E-03 | ZFP30 | -0.55 | 3.29E-03 | 0.0262 |
| KCNN4 | 1.47 | 8.43E-08 | 2.34E-05 | SEMA6A | -0.9 | 3.63E-05 | 1.26E-03 | RARRES1 | 1.01 | 4.84E-06 | 3.26E-04 | HPDL | -0.55 | 4.67E-03 | 0.0337 |
| TFAP2C | 1.45 | 3.41E-05 | 1.20E-03 | NCKAP5 | -0.85 | 1.63E-04 | 3.48E-03 | TIMP1 | 1 | 2.74E-11 | 1.14E-07 | P2RX7 | -0.55 | 5.24E-03 | 0.0366 |
| MIA | 1.44 | 5.99E-07 | 8.05E-05 | MTURN | -0.84 | 1.63E-07 | 3.35E-05 | SMIM3 | 1 | 4.79E-08 | 1.67E-05 | ZNF697 | -0.54 | 1.81E-04 | 0.00367 |
| MMP9 | 1.42 | 6.73E-07 | 8.86E-05 | SLC6A15 | -0.84 | 2.25E-03 | 0.0204 | FAM20A | 1 | 2.66E-06 | 2.20E-04 | PTK7 | -0.54 | 1.91E-03 | 0.0182 |
| CMBL | 1.42 | 2.30E-06 | 2.06E-04 | EGR1 | -0.82 | 3.23E-06 | 2.49E-04 | P3H2 | 1 | 6.24E-04 | 8.43E-03 | ARSG | -0.54 | 5.77E-03 | 0.0389 |
| ITGB3 | 1.4 | 5.00E-06 | 3.31E-04 | TUBB4A | -0.82 | 9.37E-04 | 0.0111 | TPD52L1 | 1 | 6.84E-04 | 9.00E-03 | ABL2 | -0.53 | 1.31E-06 | 1.41E-04 |
| SPP1 | 1.39 | 9.46E-08 | 2.47E-05 | IRS1 | -0.78 | 2.42E-06 | 2.10E-04 | DYNLT3 | 0.99 | 1.18E-06 | 1.32E-04 | BCL7A | -0.53 | 2.89E-05 | 1.06E-03 |
| MAOB | 1.37 | 8.30E-05 | 2.19E-03 | DUSP8 | -0.78 | 7.11E-05 | 1.97E-03 | COL13A1 | 0.99 | 1.80E-05 | 8.05E-04 | ZBED3 | -0.53 | 1.18E-04 | 2.75E-03 |
| PTPRZ1 | 1.36 | 6.55E-05 | 1.86E-03 | CABLES1 | -0.78 | 6.33E-04 | 8.51E-03 | CREB5 | 0.99 | 8.26E-05 | 2.18E-03 | PPM1H | -0.53 | 5.03E-03 | 0.0355 |
| SRPX2 | 1.35 | 1.39E-07 | 3.16E-05 | CELSR3 | -0.77 | 2.46E-05 | 9.67E-04 | CHST6 | 0.97 | 3.76E-07 | 5.88E-05 | KLF11 | -0.52 | 1.50E-09 | 1.42E-06 |
| FMOD | 1.33 | 3.42E-07 | 5.53E-05 | KCNAB2 | -0.77 | 3.74E-04 | 6.00E-03 | POSTN | 0.97 | 2.09E-04 | 4.05E-03 | LZTS3 | -0.52 | 2.89E-05 | 1.06E-03 |
| CHL1 | 1.3 | 2.86E-05 | 1.06E-03 | NINL | -0.75 | 1.62E-05 | 7.67E-04 | OPLAH | 0.97 | 7.19E-04 | 9.34E-03 | FAM86B1 | -0.52 | 1.38E-03 | 0.0145 |
| C1QTNF3 | 1.29 | 8.93E-04 | 0.0108 | EFHD1 | -0.75 | 4.54E-03 | 0.0331 | HFE | 0.96 | 1.50E-05 | 7.31E-04 | ACVR2B | -0.51 | 2.95E-05 | 1.07E-03 |
| PRR4 | 1.28 | 9.53E-09 | 5.10E-06 | CAPN3 | -0.74 | 5.92E-03 | 0.0397 | EMILIN1 | 0.96 | 2.44E-05 | 9.64E-04 | NUTM2A | -0.51 | 3.90E-05 | 1.32E-03 |
| TRPV4 | 1.27 | 2.21E-07 | 4.24E-05 | TPCN2 | -0.72 | 4.42E-06 | 3.05E-04 | SKAP1 | 0.96 | 1.74E-03 | 0.0171 | ZNF571 | -0.51 | 8.95E-05 | 2.27E-03 |
| TMEM163 | 1.27 | 4.76E-04 | 7.04E-03 | ATP8A1 | -0.72 | 3.15E-03 | 0.0256 | MT2A | 0.95 | 5.21E-07 | 7.40E-05 | WTIP | -0.51 | 4.15E-04 | 6.42E-03 |
| CEMIP | 1.25 | 3.35E-06 | 2.50E-04 | GCNT2 | -0.7 | 4.63E-04 | 6.88E-03 | TSPAN13 | 0.95 | 3.50E-06 | 2.53E-04 | PLEKHG3 | -0.51 | 4.42E-04 | 6.64E-03 |
| GPC6 | 1.22 | 1.85E-05 | 8.19E-04 | PAG1 | -0.69 | 1.13E-05 | 6.05E-04 | SNED1 | 0.95 | 5.20E-05 | 1.59E-03 | PARD6G | -0.5 | 5.83E-06 | 3.74E-04 |
| NNMT | 1.2 | 2.66E-08 | 1.07E-05 | SNAI2 | -0.69 | 5.97E-05 | 1.76E-03 | PRTFDC1 | 0.95 | 2.48E-04 | 4.51E-03 | AMOTL2 | -0.5 | 1.92E-05 | 8.44E-04 |
| SLC2A10 | 1.2 | 1.30E-07 | 3.09E-05 | NR4A3 | -0.69 | 4.02E-03 | 0.0302 | SPOCD1 | 0.95 | 3.21E-04 | 5.40E-03 | MYC | -0.5 | 1.33E-04 | 3.00E-03 |
| GJB2 | 1.19 | 9.79E-04 | 0.0114 | C16ORF74 | -0.68 | 3.89E-03 | 0.0295 | KCNJ10 | 0.95 | 4.26E-03 | 0.0315 | EPM2A | -0.49 | 8.48E-06 | 4.91E-04 |
| FGF2 | 1.18 | 1.28E-05 | 6.50E-04 | TDRP | -0.67 | 1.08E-03 | 0.0123 | MICALL2 | 0.94 | 8.18E-08 | 2.33E-05 | NEK3 | -0.49 | 1.79E-04 | 3.65E-03 |
| STRA6 | 1.18 | 1.45E-04 | 3.19E-03 | FAM53B | -0.65 | 6.09E-08 | 1.95E-05 | PCOLCE | 0.94 | 5.26E-05 | 1.60E-03 | PRKX | -0.49 | 5.80E-04 | 8.02E-03 |
| CHI3L1 | 1.17 | 3.04E-04 | 5.19E-03 | ZNF607 | -0.65 | 1.04E-03 | 0.0119 | HOXB2 | 0.94 | 6.97E-05 | 1.95E-03 | CYP2U1 | -0.49 | 1.37E-03 | 0.0144 |
| FGFBP2 | 1.13 | 1.14E-04 | 2.70E-03 | PER3 | -0.64 | 2.57E-07 | 4.57E-05 | ALDH1A3 | 0.94 | 1.90E-03 | 0.0182 | Mar-00 | -0.48 | 1.97E-06 | 1.87E-04 |
| CA12 | 1.13 | 2.35E-04 | 4.38E-03 | LRIG3 | -0.64 | 4.36E-05 | 1.41E-03 | IL27RA | 0.93 | 7.60E-07 | 9.69E-05 | ATP7A | -0.48 | 2.27E-05 | 9.39E-04 |
| IGFBP3 | 1.12 | 1.31E-07 | 3.09E-05 | CEACAM19 | -0.64 | 9.76E-04 | 0.0114 | CITED4 | 0.93 | 1.15E-05 | 6.09E-04 | ZKSCAN8 | -0.48 | 6.00E-05 | 1.77E-03 |
| LRATD2 | 1.11 | 3.36E-06 | 2.50E-04 | SBK1 | -0.64 | 2.86E-03 | 0.0241 | SULF1 | 0.93 | 2.54E-05 | 9.78E-04 | STK32C | -0.48 | 4.24E-04 | 6.49E-03 |
| CFI | 1.11 | 5.01E-06 | 3.31E-04 | ATP6V1G2 | -0.62 | 7.56E-05 | 2.06E-03 | SORL1 | 0.93 | 7.37E-05 | 2.04E-03 | LCOR | -0.48 | 8.16E-04 | 0.0102 |
| CSPG5 | 1.11 | 2.48E-05 | 9.70E-04 | TFAP2E | -0.61 | 1.45E-03 | 0.015 | COL9A2 | 0.93 | 1.92E-04 | 3.83E-03 | RAC3 | -0.48 | 5.45E-03 | 0.0375 |
| CNIH3 | 1.1 | 3.93E-05 | 1.32E-03 | AATBC | -0.61 | 5.45E-03 | 0.0375 | PPP1R9A | 0.93 | 8.39E-04 | 0.0104 | LRIG2 | -0.47 | 2.12E-08 | 8.83E-06 |
| FAM131B | 1.1 | 1.02E-04 | 2.48E-03 | RTTN | -0.6 | 2.08E-05 | 8.83E-04 | LOXL3 | 0.93 | 1.25E-03 | 0.0136 | KLF6 | -0.47 | 6.51E-06 | 4.05E-04 |
| IGFBP2 | 1.09 | 2.82E-05 | 1.06E-03 | SYDE2 | -0.6 | 1.89E-03 | 0.0181 | MISP3 | 0.92 | 4.71E-06 | 3.20E-04 | NLN | -0.47 | 1.12E-05 | 6.05E-04 |
| PRRX1 | 1.08 | 9.88E-07 | 1.15E-04 | CPEB2 | -0.59 | 2.03E-05 | 8.76E-04 | CTHRC1 | 0.92 | 5.50E-06 | 3.58E-04 | FBXL2 | -0.47 | 1.65E-04 | 3.50E-03 |
| ELOVL2 | 1.08 | 1.14E-03 | 0.0128 | BCL2 | -0.59 | 4.64E-05 | 1.47E-03 | PDGFRL | 0.92 | 1.26E-05 | 6.39E-04 | FRAT2 | -0.46 | 1.19E-08 | 5.49E-06 |
| MRC2 | 1.07 | 9.26E-07 | 1.12E-04 | DAPK1 | -0.59 | 4.84E-05 | 1.51E-03 | IGFBP6 | 0.91 | 6.21E-06 | 3.90E-04 | RPS18 | -0.46 | 2.57E-06 | 2.17E-04 |
| PPL | 1.07 | 2.02E-04 | 3.97E-03 | GRK3 | -0.59 | 4.24E-04 | 6.49E-03 | FAP | 0.91 | 9.20E-06 | 5.25E-04 | RHOBTB1 | -0.46 | 6.22E-05 | 1.80E-03 |
| PDLIM4 | 1.07 | 1.50E-03 | 0.0154 | LIMS2 | -0.59 | 1.18E-03 | 0.0131 | OCIAD2 | 0.91 | 5.35E-04 | 7.58E-03 | TAF4B | -0.46 | 2.24E-04 | 4.22E-03 |
| STC1 | 1.06 | 3.83E-06 | 2.71E-04 | KLF5 | -0.59 | 2.60E-03 | 0.0226 | CAVIN3 | 0.9 | 1.22E-07 | 3.05E-05 | R3HCC1L | -0.45 | 3.99E-06 | 2.80E-04 |
| SLC26A4 | 1.06 | 5.50E-05 | 1.65E-03 | MCC | -0.58 | 3.79E-04 | 6.04E-03 | COPZ2 | 0.9 | 1.00E-06 | 1.15E-04 | TRAK2 | -0.45 | 1.70E-05 | 7.79E-04 |
| DNALI1 | 1.06 | 4.45E-04 | 6.67E-03 | ATP11A | -0.58 | 9.94E-04 | 0.0116 | CHST1 | 0.9 | 2.63E-06 | 2.19E-04 | SLC7A1 | -0.45 | 1.92E-05 | 8.44E-04 |
| SCUBE2 | 1.06 | 2.39E-03 | 0.0213 | IER2 | -0.57 | 2.56E-09 | 2.00E-06 | GFPT2 | 0.9 | 1.62E-05 | 7.67E-04 | ZNF251 | -0.45 | 2.47E-05 | 9.68E-04 |
Supplementary table S2: 2071 genes were differentially expressed in BRAFV600E/wildtype patient melanomas in data extracted from the TCGA firehouse legacy dataset. Top 100 genes differentially expressed (ranked according to Log2 ratio)

## Slide 7
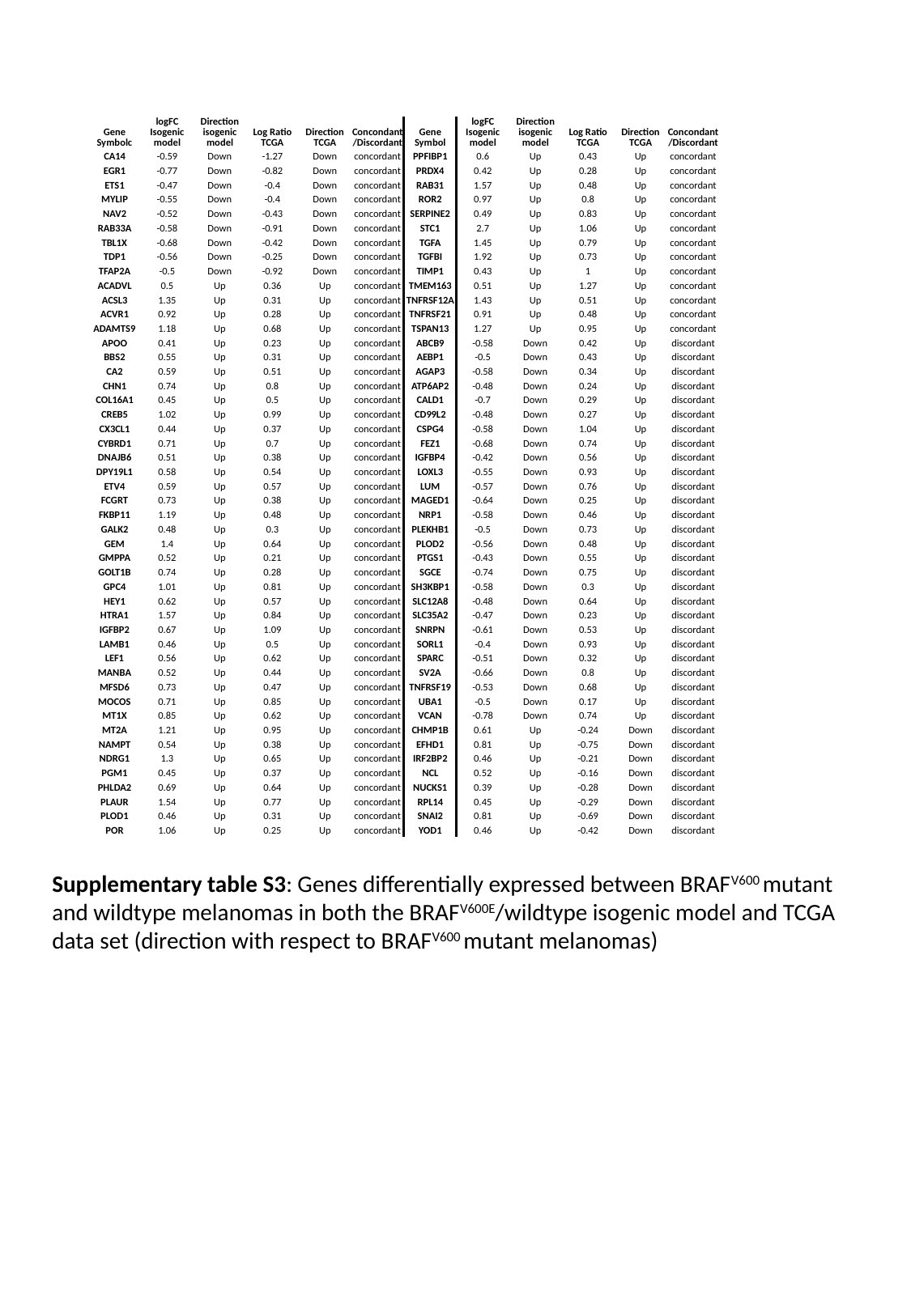

| Gene Symbolc | logFC Isogenic model | Direction isogenic model | Log Ratio TCGA | Direction TCGA | Concondant/Discordant | Gene Symbol | logFC Isogenic model | Direction isogenic model | Log Ratio TCGA | Direction TCGA | Concondant/Discordant |
| --- | --- | --- | --- | --- | --- | --- | --- | --- | --- | --- | --- |
| CA14 | -0.59 | Down | -1.27 | Down | concordant | PPFIBP1 | 0.6 | Up | 0.43 | Up | concordant |
| EGR1 | -0.77 | Down | -0.82 | Down | concordant | PRDX4 | 0.42 | Up | 0.28 | Up | concordant |
| ETS1 | -0.47 | Down | -0.4 | Down | concordant | RAB31 | 1.57 | Up | 0.48 | Up | concordant |
| MYLIP | -0.55 | Down | -0.4 | Down | concordant | ROR2 | 0.97 | Up | 0.8 | Up | concordant |
| NAV2 | -0.52 | Down | -0.43 | Down | concordant | SERPINE2 | 0.49 | Up | 0.83 | Up | concordant |
| RAB33A | -0.58 | Down | -0.91 | Down | concordant | STC1 | 2.7 | Up | 1.06 | Up | concordant |
| TBL1X | -0.68 | Down | -0.42 | Down | concordant | TGFA | 1.45 | Up | 0.79 | Up | concordant |
| TDP1 | -0.56 | Down | -0.25 | Down | concordant | TGFBI | 1.92 | Up | 0.73 | Up | concordant |
| TFAP2A | -0.5 | Down | -0.92 | Down | concordant | TIMP1 | 0.43 | Up | 1 | Up | concordant |
| ACADVL | 0.5 | Up | 0.36 | Up | concordant | TMEM163 | 0.51 | Up | 1.27 | Up | concordant |
| ACSL3 | 1.35 | Up | 0.31 | Up | concordant | TNFRSF12A | 1.43 | Up | 0.51 | Up | concordant |
| ACVR1 | 0.92 | Up | 0.28 | Up | concordant | TNFRSF21 | 0.91 | Up | 0.48 | Up | concordant |
| ADAMTS9 | 1.18 | Up | 0.68 | Up | concordant | TSPAN13 | 1.27 | Up | 0.95 | Up | concordant |
| APOO | 0.41 | Up | 0.23 | Up | concordant | ABCB9 | -0.58 | Down | 0.42 | Up | discordant |
| BBS2 | 0.55 | Up | 0.31 | Up | concordant | AEBP1 | -0.5 | Down | 0.43 | Up | discordant |
| CA2 | 0.59 | Up | 0.51 | Up | concordant | AGAP3 | -0.58 | Down | 0.34 | Up | discordant |
| CHN1 | 0.74 | Up | 0.8 | Up | concordant | ATP6AP2 | -0.48 | Down | 0.24 | Up | discordant |
| COL16A1 | 0.45 | Up | 0.5 | Up | concordant | CALD1 | -0.7 | Down | 0.29 | Up | discordant |
| CREB5 | 1.02 | Up | 0.99 | Up | concordant | CD99L2 | -0.48 | Down | 0.27 | Up | discordant |
| CX3CL1 | 0.44 | Up | 0.37 | Up | concordant | CSPG4 | -0.58 | Down | 1.04 | Up | discordant |
| CYBRD1 | 0.71 | Up | 0.7 | Up | concordant | FEZ1 | -0.68 | Down | 0.74 | Up | discordant |
| DNAJB6 | 0.51 | Up | 0.38 | Up | concordant | IGFBP4 | -0.42 | Down | 0.56 | Up | discordant |
| DPY19L1 | 0.58 | Up | 0.54 | Up | concordant | LOXL3 | -0.55 | Down | 0.93 | Up | discordant |
| ETV4 | 0.59 | Up | 0.57 | Up | concordant | LUM | -0.57 | Down | 0.76 | Up | discordant |
| FCGRT | 0.73 | Up | 0.38 | Up | concordant | MAGED1 | -0.64 | Down | 0.25 | Up | discordant |
| FKBP11 | 1.19 | Up | 0.48 | Up | concordant | NRP1 | -0.58 | Down | 0.46 | Up | discordant |
| GALK2 | 0.48 | Up | 0.3 | Up | concordant | PLEKHB1 | -0.5 | Down | 0.73 | Up | discordant |
| GEM | 1.4 | Up | 0.64 | Up | concordant | PLOD2 | -0.56 | Down | 0.48 | Up | discordant |
| GMPPA | 0.52 | Up | 0.21 | Up | concordant | PTGS1 | -0.43 | Down | 0.55 | Up | discordant |
| GOLT1B | 0.74 | Up | 0.28 | Up | concordant | SGCE | -0.74 | Down | 0.75 | Up | discordant |
| GPC4 | 1.01 | Up | 0.81 | Up | concordant | SH3KBP1 | -0.58 | Down | 0.3 | Up | discordant |
| HEY1 | 0.62 | Up | 0.57 | Up | concordant | SLC12A8 | -0.48 | Down | 0.64 | Up | discordant |
| HTRA1 | 1.57 | Up | 0.84 | Up | concordant | SLC35A2 | -0.47 | Down | 0.23 | Up | discordant |
| IGFBP2 | 0.67 | Up | 1.09 | Up | concordant | SNRPN | -0.61 | Down | 0.53 | Up | discordant |
| LAMB1 | 0.46 | Up | 0.5 | Up | concordant | SORL1 | -0.4 | Down | 0.93 | Up | discordant |
| LEF1 | 0.56 | Up | 0.62 | Up | concordant | SPARC | -0.51 | Down | 0.32 | Up | discordant |
| MANBA | 0.52 | Up | 0.44 | Up | concordant | SV2A | -0.66 | Down | 0.8 | Up | discordant |
| MFSD6 | 0.73 | Up | 0.47 | Up | concordant | TNFRSF19 | -0.53 | Down | 0.68 | Up | discordant |
| MOCOS | 0.71 | Up | 0.85 | Up | concordant | UBA1 | -0.5 | Down | 0.17 | Up | discordant |
| MT1X | 0.85 | Up | 0.62 | Up | concordant | VCAN | -0.78 | Down | 0.74 | Up | discordant |
| MT2A | 1.21 | Up | 0.95 | Up | concordant | CHMP1B | 0.61 | Up | -0.24 | Down | discordant |
| NAMPT | 0.54 | Up | 0.38 | Up | concordant | EFHD1 | 0.81 | Up | -0.75 | Down | discordant |
| NDRG1 | 1.3 | Up | 0.65 | Up | concordant | IRF2BP2 | 0.46 | Up | -0.21 | Down | discordant |
| PGM1 | 0.45 | Up | 0.37 | Up | concordant | NCL | 0.52 | Up | -0.16 | Down | discordant |
| PHLDA2 | 0.69 | Up | 0.64 | Up | concordant | NUCKS1 | 0.39 | Up | -0.28 | Down | discordant |
| PLAUR | 1.54 | Up | 0.77 | Up | concordant | RPL14 | 0.45 | Up | -0.29 | Down | discordant |
| PLOD1 | 0.46 | Up | 0.31 | Up | concordant | SNAI2 | 0.81 | Up | -0.69 | Down | discordant |
| POR | 1.06 | Up | 0.25 | Up | concordant | YOD1 | 0.46 | Up | -0.42 | Down | discordant |
Supplementary table S3: Genes differentially expressed between BRAFV600 mutant and wildtype melanomas in both the BRAFV600E/wildtype isogenic model and TCGA data set (direction with respect to BRAFV600 mutant melanomas)

## Slide 8
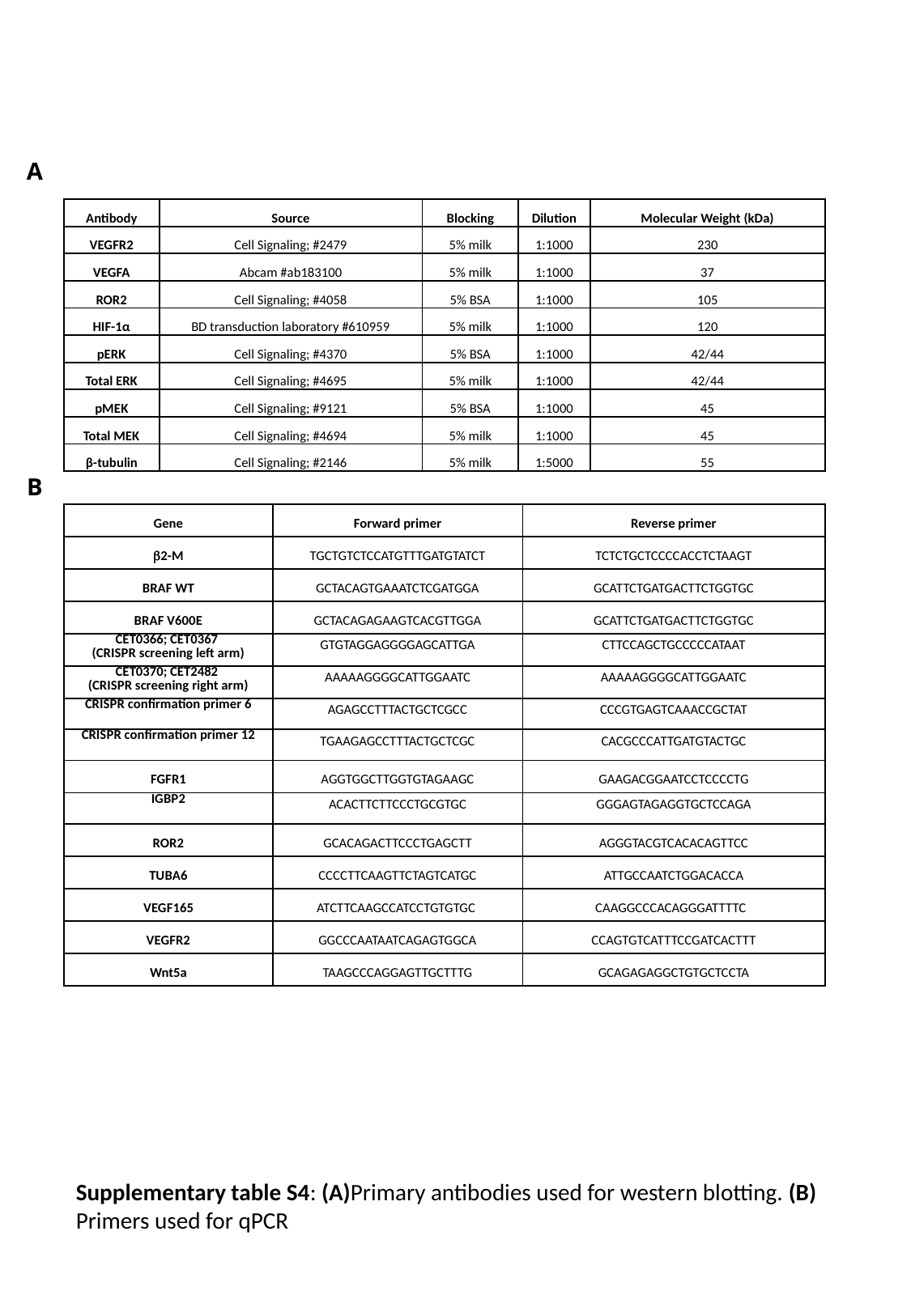

A
| Antibody | Source | Blocking | Dilution | Molecular Weight (kDa) |
| --- | --- | --- | --- | --- |
| VEGFR2 | Cell Signaling; #2479 | 5% milk | 1:1000 | 230 |
| VEGFA | Abcam #ab183100 | 5% milk | 1:1000 | 37 |
| ROR2 | Cell Signaling; #4058 | 5% BSA | 1:1000 | 105 |
| HIF-1α | BD transduction laboratory #610959 | 5% milk | 1:1000 | 120 |
| pERK | Cell Signaling; #4370 | 5% BSA | 1:1000 | 42/44 |
| Total ERK | Cell Signaling; #4695 | 5% milk | 1:1000 | 42/44 |
| pMEK | Cell Signaling; #9121 | 5% BSA | 1:1000 | 45 |
| Total MEK | Cell Signaling; #4694 | 5% milk | 1:1000 | 45 |
| β-tubulin | Cell Signaling; #2146 | 5% milk | 1:5000 | 55 |
B
| Gene | Forward primer | Reverse primer |
| --- | --- | --- |
| β2-M | TGCTGTCTCCATGTTTGATGTATCT | TCTCTGCTCCCCACCTCTAAGT |
| BRAF WT | GCTACAGTGAAATCTCGATGGA | GCATTCTGATGACTTCTGGTGC |
| BRAF V600E | GCTACAGAGAAGTCACGTTGGA | GCATTCTGATGACTTCTGGTGC |
| CET0366; CET0367 (CRISPR screening left arm) | GTGTAGGAGGGGAGCATTGA | CTTCCAGCTGCCCCCATAAT |
| CET0370; CET2482 (CRISPR screening right arm) | AAAAAGGGGCATTGGAATC | AAAAAGGGGCATTGGAATC |
| CRISPR confirmation primer 6 | AGAGCCTTTACTGCTCGCC | CCCGTGAGTCAAACCGCTAT |
| CRISPR confirmation primer 12 | TGAAGAGCCTTTACTGCTCGC | CACGCCCATTGATGTACTGC |
| FGFR1 | AGGTGGCTTGGTGTAGAAGC | GAAGACGGAATCCTCCCCTG |
| IGBP2 | ACACTTCTTCCCTGCGTGC | GGGAGTAGAGGTGCTCCAGA |
| ROR2 | GCACAGACTTCCCTGAGCTT | AGGGTACGTCACACAGTTCC |
| TUBA6 | CCCCTTCAAGTTCTAGTCATGC | ATTGCCAATCTGGACACCA |
| VEGF­165 | ATCTTCAAGCCATCCTGTGTGC | CAAGGCCCACAGGGATTTTC |
| VEGFR2 | GGCCCAATAATCAGAGTGGCA | CCAGTGTCATTTCCGATCACTTT |
| Wnt5a | TAAGCCCAGGAGTTGCTTTG | GCAGAGAGGCTGTGCTCCTA |
Supplementary table S4: (A)Primary antibodies used for western blotting. (B) Primers used for qPCR
